# Mitogenomics of *Culex pipiens* Form Pipiens From the Turkish Black Sea Region Reveals Structural Conservation and Phylogenetic Complexity

**DOI:** 10.1002/ece3.74085

**Published:** 2026-08-02

**Authors:** Ahsen Meliha Toroslu, Alparslan Yildirim, Gokmen Zafer Pekmezci, Osman Ibis, Samba Deguene Diop, Batuhan Askim Arslanhan, Saffet Teber, Alina Denis Kizgin, Simge Sahin, Filiz Gunay, Nathan Burkett‐Cadena, Lindsay Campbell, Abdullah Inci, Barry Wilmer Alto

**Affiliations:** ^1^ Department of Parasitology, Faculty of Veterinary Medicine Ondokuz Mayis University Samsun Türkiye; ^2^ Department of Parasitology, Faculty of Veterinary Medicine Erciyes University Kayseri Türkiye; ^3^ Department of Preclinical Sciences, Faculty of Veterinary Medicine Ondokuz Mayis University Samsun Türkiye; ^4^ Department of Agricultural Biotechnology, Faculty of Agriculture Erciyes University Kayseri Türkiye; ^5^ Florida Medical Entomology Laboratory, IFAS University of Florida Vero Beach Florida USA; ^6^ Department of Biology, Ecology Section Hacettepe University Ankara Turkiye; ^7^ Department of Entomology and Nematology, IFAS University of Florida Gainesville Florida USA

**Keywords:** Black Sea region, complete mitochondrial genome, *Culex pipiens* complex, *Culex pipiens* form pipiens, introgression, mitogenomics, phylogenomics

## Abstract

The *Culex pipiens* complex includes major mosquito vectors involved in the transmission of West Nile virus and filarial nematodes. In Türkiye, particularly in the Black Sea region, members of this complex are widely distributed, yet mitogenome‐scale data from local populations remain limited. Here, we sequenced, assembled, and annotated the complete mitochondrial genomes of five *Culex pipiens* form pipiens isolates collected from different provinces along the Turkish Black Sea coast. Following the current taxonomic interpretation, subspecies‐level names were avoided, *Cx. pallens* was treated as a species, and molestus was treated as a form of *Cx. pipiens*. The newly generated mitogenomes were highly conserved, ranging from 15,602 to 15,604 bp, and each contained the typical set of 37 mitochondrial genes with a strong A + T bias (~78.2%). Comparative analyses showed a conserved gene order and a characteristic culicid mitogenome architecture. Expanded comparisons across the broader *Cx. pipiens* complex dataset showed that mitochondrial diversity was concentrated in selected loci, particularly COX1, ND2, COX3, CYTB, ND5, and 12S rRNA, as well as in terminal regions associated with the A + T‐rich control region. Phylogenetic analyses based on the concatenated sequences of 13 mitochondrial protein‐coding genes and two rRNA genes showed that the Turkish isolates do not form a single exclusive mitochondrial lineage, but are distributed across different parts of the ingroup together with other members of the *Cx. pipiens* complex, including *Cx. pipiens* form molestus, *Cx. pallens*, and *Cx. quinquefasciatus*. These findings support the view that mitochondrial genomes in this complex primarily reflect maternal lineage history and may be influenced by introgression and incomplete lineage sorting rather than sharply discrete taxonomic or form‐level boundaries. Overall, the mitogenomic resources generated in this study provide a useful regional reference for future comparative and evolutionary studies of the *Cx. pipiens* complex.

## Graphical abstract

Complete mitochondrial genomes of five Turkish *Culex pipiens* form pipiens isolates from the Black Sea region revealed highly conserved mitogenome architecture. Expanded comparisons across the broader *Cx. pipiens* complex showed that mitochondrial diversity was concentrated in selected loci and terminal regions associated with the A + T‐rich control region. Phylogenetic analysis showed that the Turkish isolates did not form a single exclusive mitochondrial lineage, supporting maternal lineage complexity within the broader complex.

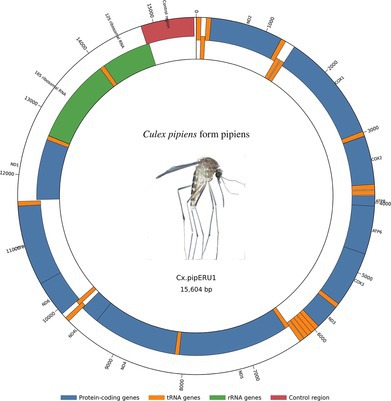

## 1 Introduction

Mosquitoes of the genus *Culex* are among the most important vectors of human and veterinary pathogens worldwide, and the *Culex pipiens* complex is of particular concern because of its broad distribution, ecological plasticity, and role in the transmission of arboviruses, avian *Plasmodium* parasites, and filarial parasites (Becker et al. 2010; Farajollahi et al. 2011; Chancey et al. 2015). In Europe, the Mediterranean Basin, and North America, members of the *Cx. pipiens* complex have frequently been implicated in the circulation of West Nile virus (WNV) and Usutu virus (USUV), and their epidemiological importance may increase in regions where ecological heterogeneity, urbanization, and climate‐driven range shifts intensify contact among avian reservoirs, mammalian hosts, and vector populations (Engler et al. 2013; Fortuna et al. 2015; Zannoli and Sambri 2019; Saarman et al. 2025). This issue is particularly relevant to Türkiye, a major migratory bird corridor in which WNV circulation and human infections have already been documented (Kalaycıoğlu et al. 2012).

The *Cx. pipiens* complex has long posed a taxonomic and evolutionary challenge because its members are morphologically similar yet differ substantially in host preference, organismal ecology, physiology, and vectorial capacity (Vinogradova 2000; Harbach 2012). Current taxonomic perspectives recognize globally distributed taxa such as *Cx. pipiens* and *Cx. quinquefasciatus*, the species *Cx. pallens*, and regionally restricted species such as *Cx. australicus* within the broader evolutionary framework of the complex (Harbach 2012; Aardema et al. 2022; Harbach and Wilkerson 2023). Harbach and Wilkerson (2023) rejected the use of subspecies in mosquito classification, re‐established *Cx. pallens* as a species, and treated molestus as a physiological variant, or molestus form, of *Cx. pipiens*. Within *Cx. pipiens*, two major ecological forms are widely recognized: the pipiens form and the molestus form. The pipiens form is generally associated with aboveground habitats, winter diapause, eurygamy, and predominantly ornithophilic feeding behavior, whereas the molestus form is typically linked to subterranean or enclosed human‐made habitats, lacks diapause, mates in confined spaces, often shows autogeny, and has a stronger tendency toward mammal‐feeding (Kent et al. 2007; Haba and McBride 2022; Haba et al. 2025).

Because morphology alone is insufficient for reliable identification within this complex, molecular tools based on nuclear markers have become central to distinguishing taxa, biotypes, and their hybrids. The CQ11 microsatellite locus and the acetylcholinesterase‐2 gene (ace‐2) have been widely used to discriminate *Cx. pipiens* from *Cx. quinquefasciatus*, as well as to distinguish the pipiens and molestus forms in regions where they occur sympatrically (Smith and Fonseca 2004; Alvial et al. 2024). This distinction is epidemiologically significant because hybridization within the complex, particularly between pipiens and molestus forms or between *Cx. pipiens* and *Cx. quinquefasciatus*, can modify host‐feeding behavior and increase the risk of pathogen spillover from avian reservoirs to humans (Fonseca et al. 2004; Saarman et al. 2025). In Türkiye, DNA barcoding and region‐specific microsatellite analyses have shown that *Cx. pipiens* form pipiens, *Cx. pipiens* form molestus, *Cx. quinquefasciatus*, and their hybrids are broadly distributed, including in the Black Sea region (Günay et al. 2015; Şahingöz Demirpolat et al. 2018; Akıner et al. 2022). Nevertheless, populations of the *Cx. pipiens* complex in Türkiye remain insufficiently characterized at the level of complete mitochondrial genomes.

Complete mitochondrial genomes provide a more comprehensive comparative framework than short barcode fragments for assessing genome organization, nucleotide composition, codon usage patterns, RNA structure, and maternal evolutionary relationships. In insects, the mitogenome typically comprises 13 protein‐coding genes, 22 transfer RNAs, two ribosomal RNAs, and a control region, and it has proven informative in comparative and phylogenetic studies across diverse taxa (Boore 1999; Simon et al. 2006). In mosquitoes, mitogenomic analyses have been widely applied to characterize compositional bias, codon usage patterns, RNA features, and maternal phylogenetic structure (Behura et al. 2011; Luo et al. 2016). However, mitochondrial phylogenies within the *Cx. pipiens* complex should be interpreted with caution. Recent genomic studies indicate that incomplete lineage sorting, historical admixture, and endosymbiont‐associated selective sweeps can obscure mitochondrial differentiation among taxa and biotypes. As a result, mitogenome‐based phylogenies may reflect shared maternal ancestry rather than fully resolved evolutionary boundaries or reproductive isolation (Yurchenko et al. 2020; Aardema et al. 2022).

Despite the medical and ecological importance of the *Cx. pipiens* complex in Türkiye, complete mitochondrial genome data remain scarce, particularly for populations in the Black Sea region. This gap is important because Türkiye lies at the intersection of European, Asian, and Middle Eastern faunal regions and includes major migratory bird corridors relevant to vector‐borne pathogen circulation. Complete mitogenomes from this region provide a broader maternal‐lineage reference than short barcode fragments, allowing Turkish populations to be evaluated within the wider diversity of the *Cx. pipiens* complex and providing a baseline for future comparative, phylogeographic, and surveillance‐oriented studies. In this study, we characterized and compared the complete mitochondrial genomes of five *Cx. pipiens* form pipiens isolates collected from this region. We analyzed genome organization, nucleotide composition, and sequence variation among isolates, and used concatenated mitochondrial gene datasets to infer their phylogenetic relationships within the broader *Cx. pipiens* complex. Our aim was to establish a regional mitogenomic framework for Turkish populations and to evaluate how these isolates contribute to the maternal evolutionary diversity of this medically important vector complex.

## 2 Materials and Methods

### 2.1 Mosquito Material, Identification, and Comparative Mitogenome Dataset

**FIGURE 1:**
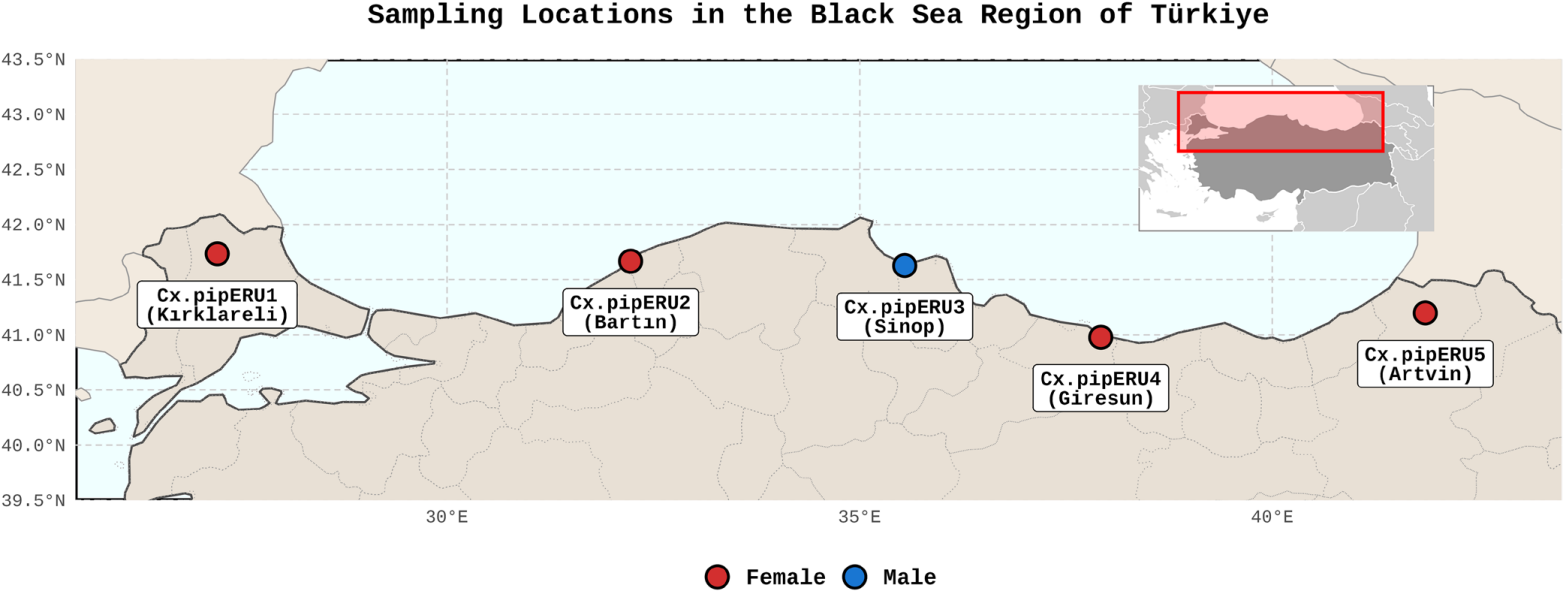
Geographic distribution of the *Cx. pipiens* form pipiens isolates analyzed in this study. The main map illustrates the specific sampling sites across five provinces (Kırklareli, Bartın, Sinop, Giresun, and Artvin) positioned along the Black Sea coast of Türkiye, including provincial borders. Colored circles denote the sex of the sequenced adult specimens (red for females, blue for the single male isolate). The inset map provides a national overview, with the red rectangular frame demarcating the focused study area.

The five *Culex pipiens* specimens analyzed in this study were selected from the samples originated from field surveys conducted between May and September 2024 for a project entitled *Enhancing arbovirus surveillance and risk management in the public health systems of Georgia, Turkey, and Ukraine* (Grant/Award No. HDTRA12210015). Erciyes University served as the Turkish subrecipient responsible for field and laboratory activities in the Black Sea region. Briefly, ovitrap sampling was conducted at each locality using black plastic cups containing cardboard strips as oviposition substrates and grass infusion as an attractant (Sant'ana et al. 2006). Ovitraps were checked and replaced every 6 days. Cardboard strips containing *Aedes* eggs and infusion water containing *Aedes* and *Culex* larvae were transported alive to an insectary at Erciyes University for species identification, rearing, and further processing of samples. During the 2024 surveillance period, ovitrap‐derived eggs and larvae were collected from five provinces in the Black Sea region of Türkiye, namely Kirklareli, Bartin, Sinop, Giresun, and Artvin. The geographic distribution of the sampling sites is shown in Figure 1, and detailed locality information is provided in Table 1.

**TABLE 1:** Complete mitochondrial genome dataset used in this study, with taxon names updated according to current taxonomic interpretation.

Seq no	Taxon/sample name	Isolate/voucher	GenBank accession	Genome length (bp)	Geographic location
1	*Culex pipiens* form pipiens	Cx.pipERU1 (female)	PZ220808	15,604	Kırklareli, Türkiye (41.734879, 27.218442)
2	*Culex pipiens* form pipiens	Cx.pipERU2 (female)	PZ220809	15,602	Bartın, Türkiye (41.667346, 32.225630)
3	*Culex pipiens* form pipiens	Cx.pipERU3 (male)	PZ220810	15,604	Sinop, Türkiye (41.627462, 35.545902)
4	*Culex pipiens* form pipiens	Cx.pipERU4 (female)	PZ220811	15,603	Giresun, Türkiye (40.976936, 37.922374)
5	*Culex pipiens* form pipiens	Cx.pipERU5 (female)	PZ220812	15,603	Artvin, Türkiye (41.197810, 41.852853)
6	*Culex pipiens* form pipiens	—	HQ724614.1 (NC_015079)	14,856	Tunisia
7	*Culex pipiens* form pipiens	—	HQ724615.1	14,856	Greece: Crete
8	*Culex pipiens* form pipiens	—	OQ533057.1	14,856	Iraq
9	*Culex pipiens* form molestus	MI149	MN389459.1	15,584	Australia: Carwoola, NSW
10	*Culex pipiens* form molestus	W82	MN389460.1	15,584	Australia: Sydney Olympic Park, NSW
11	*Culex pipiens* form molestus	—	PQ584792.2	15,636	South Korea
12	*Culex pallens*	—	KT851543.1	15,617	China: Shaanxi
13	*Culex quinquefasciatus*	—	HQ724617.1	14,856	USA: California
14	*Culex quinquefasciatus*	—	MK575480.1	15,286	Brazil
15	*Culex quinquefasciatus*	NIMS2301706	PV176466.1	15,586	British Indian Ocean Territory
16	*Culex australicus*	W03	MN389456.1	15,582	Australia: Gungahlin, ACT
17	*Culex coronator*	RS10_109	MF040162.1	15,576	Brazil: Rio Grande do Sul
18	*Culex coronator*	—	MF381691.1	15,576	Brazil
19	*Culex coronator*	ss_MIS‐14‐07	MF509890.1	15,576	Argentina
20	*Culex gelidus*	—	KX753344.1	15,600	China

*Note:* Taxon names were updated according to Harbach and Wilkerson (2023), who rejected the use of subspecies in mosquito classification, re‐established *Culex pallens* as a species, and treated molestus as a physiological/ecological form of *Cx. pipiens*. GenBank accession numbers were retained unchanged to preserve sequence traceability. Therefore, updated taxon names shown here may differ from the names originally deposited in GenBank.

The collected immature stages were reared under standard insectary conditions to obtain F1 adults. The resulting specimens were first examined morphologically and assigned to the *Culex pipiens* complex. For the present study, one isolate from each province was selected and designated as Cx.pipERU1, Cx.pipERU2, Cx.pipERU3, Cx.pipERU4, and Cx.pipERU5, respectively. Molecular confirmation and form identification were then performed using the acetylcholinesterase‐2 (ace‐2) locus and the CQ11 microsatellite marker, following the diagnostic frameworks of Malcolm et al. (1998) and Smith and Fonseca (2004). Species and form assignments were based on the expected amplification profiles and diagnostic banding patterns.

To place the newly generated Turkish mitogenomes in a broader comparative framework, a reference dataset of complete mitochondrial genomes representing the *Culex pipiens* complex was compiled from GenBank. Only complete or near complete mitogenome records with clear taxonomic or form assignment were retained. The final comparative dataset included representative sequences of *Cx. pipiens* form pipiens, *Cx. pipiens* form molestus, *Cx. pallens*, *Cx. quinquefasciatus*, and *Cx. australicus*, together with selected outgroup taxa used for phylogenetic analyses.

Accession numbers, locality information, and taxonomic identities of all comparative mitogenomes are listed in Table 1. Taxon names of GenBank‐derived reference mitogenomes were updated according to Harbach and Wilkerson (2023), while accession numbers were retained unchanged to preserve sequence traceability.

### 2.2 Genomic DNA Extraction, Library Preparation, and Next‐Generation Sequencing

Total genomic DNA was extracted individually from adult mosquito specimens using the PureLink Genomic DNA Mini Kit (Invitrogen, Thermo Fisher Scientific, Waltham, MA, USA) following the manufacturer's standard protocol. The extracted DNA was eluted in PureLink Genomic Elution Buffer within the recommended 25–200 μL volume range to ensure optimal concentration for downstream applications.

Next‐generation sequencing (NGS) was performed commercially by Gen Era Diagnostics (Istanbul, Türkiye). Following rigorous DNA quality and quantity assessments, sequencing libraries were constructed using the Illumina DNA Prep Kit (Illumina, San Diego, CA, USA). This protocol employs bead‐linked transposome chemistry, which facilitates rapid and uniform library preparation by integrating simultaneous DNA fragmentation and adapter incorporation (tagmentation), followed by PCR‐based index addition and subsequent cleanup steps.

The prepared libraries were sequenced on an Illumina NovaSeq 6000 platform, generating 150‐bp paired‐end raw reads. Across the five samples, sequencing generated 28,527,453–74,206,711 reads/fragments per sample, with an approximate target of 50 million paired‐end reads per sample. Mitochondrial read mapping produced continuous mitogenome‐wide coverage for all isolates, with mean depths ranging from 54.21× to 382.57× across samples; detailed mapping and coverage statistics are provided in Table S1. The raw sequence datasets were subsequently exported in FASTQ format for downstream quality control, trimming, and genome assembly.

### 2.3 Mitogenome Assembly and Consensus Sequence Generation

Raw paired‐end reads obtained for each sample were imported in FASTQ format into Geneious Prime 2026.0.2 (https://www.geneious.com) for read processing, reference‐guided mitochondrial genome assembly, and sequence inspection. Adapter trimming and quality filtering were performed using the BBDuk Trimming Tool implemented in Geneious Prime. In this step, Illumina adapter sequences, short reads (< 30 bp), and low‐quality bases (*Q*‐score < 20) were removed prior to assembly.

To reconstruct the mitochondrial genomes of the Turkish *Cx. pipiens* form pipiens isolates, filtered reads were first assembled using the Geneious Mapper/Map to Reference algorithm against complete mitogenomes representing members of the *Cx. pipiens* complex. Mapping was performed using the highest sensitivity/medium setting, with fine‐tuning set to iterate up to 25 times, and preliminary mitochondrial consensus sequences were generated for each isolate.

To confirm the reference‐guided assemblies and reduce possible mapping bias, the filtered reads were also assembled de novo using the GetOrganelle toolkit with default parameters (Jin et al. 2020). The filtered and trimmed reads were then remapped to the de novo mitochondrial contigs using the BBMap tool (Bushnell 2014) in Geneious Prime with the Normal Sensitivity setting to verify sequence coverage and assembly completeness. Final mitochondrial consensus sequences were generated after concordance between the reference‐guided and de novo assemblies had been confirmed.

For comparative coverage assessment under a common coordinate framework, raw paired‐end reads from all five Turkish isolates were additionally remapped to the complete mitochondrial genome of *Cx. pipiens* from Tunisia (NC_015079). These common‐reference mappings were used for comparative read‐depth visualization and summary statistics and were not used for final consensus sequence generation.

### 2.4 Genome Annotation and Mitogenome Characterization

The final mitochondrial consensus sequences obtained for the Turkish *Cx. pipiens* form pipiens isolates were annotated to identify the 13 protein‐coding genes (PCGs), 22 transfer RNA (tRNA) genes, two ribosomal RNA (rRNA) genes, and the control region (A + T‐rich region). Preliminary annotations and genome organization inferred during the Geneious‐based assembly workflow were subsequently checked and refined using the MITOS2 Web Server (Bernt et al. 2013; Donath et al. 2019), followed by BLAST‐based comparisons against annotated mosquito mitogenomes available in GenBank (Altschul et al. 1990). In addition, gene boundaries and overall gene arrangements were strictly verified by comparison with the complete *Cx. pipiens* complex mitogenomes included in Table 1. This combined strategy was used to confirm the final consensus sequences and to standardize the annotation of all Turkish isolates, in line with modern annotation workflows adopted in recent mitogenomic studies.

The putative secondary structures of the 22 tRNA and 2 rRNA genes were initially predicted by the MITOS2 pipeline. The resulting structural coordinate files were then processed using custom Python scripts within the Google Colab environment. Secondary structure visualizations were programmatically generated and plotted using the ViennaRNA package and Forgi library (Lorenz et al. 2011; Kerpedjiev et al. 2015). When automated predictions were incomplete or ambiguous, structural annotations were resolved manually by comparison with homologous *Culex* mitogenome sequences, following the comparative approach commonly used in mosquito mitogenome studies (Behura et al. 2011; Luo et al. 2016).

Mitogenome characterization included the determination of total genome length, gene order, gene orientation, lengths of coding and non‐coding regions, overlapping and intergenic spacer regions, and start and stop codon usage across all PCGs. The annotated mitogenomes were then used as the basis for downstream analyses of nucleotide composition, codon usage, RNA structural features, control‐region variation, comparative sequence divergence, and phylogenetic relationships within the *Cx. pipiens* complex. This comprehensive analytical framework aligns with comparative mosquito mitogenome research, where gene content, genome organization, codon usage, nucleotide‐skew patterns, and control‐region features are systematically characterized (Luo et al. 2016; Akintola and Hwang 2026).

### 2.5 Control Region and Comparative Sequence Variation Analyses

The control region of each Turkish *Cx. pipiens* form pipiens mitogenome was identified from the final annotations and comparatively examined with respect to its length, nucleotide composition, and structural organization. Putative tandem repeat motifs within the control region were screened using Tandem Repeats Finder (Benson 1999). Control‐region sequences were aligned using MAFFT v7 (Katoh and Standley 2013) and further compared among the Turkish isolates and across the broader *Cx. pipiens* complex reference mitogenomes included in Table 1 to evaluate repeat organization, length variation, and sequence conservation patterns. This targeted analysis was performed because size variation in the control region of closely related insect mitogenomes is often associated with tandem repeat structures and insertion–deletion (indel) polymorphisms (Luo et al. 2016; Akintola and Hwang 2026).

For comparative sequence variation analyses, pairwise sequence divergence among the five newly generated Turkish *Cx. pipiens* form pipiens mitogenomes was first assessed using complete mitogenome alignments. Pairwise p‐distances, SNP differences, and indel‐containing positions were calculated to summarize sequence divergence among the Turkish isolates.

To evaluate mitochondrial sequence variation in a broader comparative framework, an expanded *Cx. pipiens* complex dataset was then analyzed. This dataset included the five Turkish *Cx. pipiens* form pipiens mitogenomes together with representative GenBank‐derived mitogenomes of *Cx. pipiens* form pipiens, *Cx. pipiens* form molestus, *Cx. pallens*, and *Cx. quinquefasciatus*. *Cx. australicus* and the outgroup taxa used in the phylogenetic analysis were excluded from the diversity analyses because the objective was to evaluate sequence variation within the focal *Cx. pipiens* complex dataset.

Complete mitogenomes in the expanded dataset were aligned using MAFFT v7 (Katoh and Standley 2013). To evaluate the distribution of nucleotide diversity along the mitogenome, sliding‐window analysis was performed in DnaSP v6 (Rozas et al. 2017) using a window length of 200 bp and a step size of 25 bp. Nucleotide diversity (*π*) was calculated across aligned homologous positions, with ambiguous bases and gaps excluded from pairwise comparisons where applicable. Feature‐based variation statistics were calculated using the annotation coordinates of the representative Turkish mitogenome Cx.pipERU1 (PZ220808). For each annotated mitochondrial feature, variable sites, constant sites, SNP‐like columns, indel‐containing columns, and nucleotide diversity were summarized across homologous aligned positions.

To evaluate locus‐level variation, the 13 mitochondrial protein‐coding genes and two rRNA genes were extracted from each mitogenome, aligned separately using MAFFT v7, and analyzed for gene‐based nucleotide diversity. Gene‐based variation statistics included locus length, variable sites, constant sites, SNP‐like columns, indel‐containing columns, and nucleotide diversity (*π*). These analyses were used to identify mitochondrial regions contributing most strongly to sequence variation across the broader *Cx. pipiens* complex dataset. This analytical framework follows previous high‐resolution mosquito mitogenome studies in which sliding‐window and gene‐based comparisons were used to assess localized mitochondrial sequence variation, including variation in protein‐coding genes and the A + T‐rich control region (Luo et al. 2016; Akintola and Hwang 2026).

### 2.6 Phylogenetic Analyses

Phylogenetic relationships were inferred using the complete mitochondrial genome dataset assembled for this study (Table 1). This dataset included the five newly generated Turkish *Cx. pipiens* form pipiens mitogenomes, together with representative GenBank‐derived mitogenomes of *Cx. pipiens* form pipiens, *Cx. pipiens* form molestus, *Cx. pallens*, *Cx. quinquefasciatus*, and *Cx. australicus*. The outgroup set consisted of three *Cx. coronator* mitogenomes and one *Cx. gelidus* mitogenome. Taxon labels for GenBank‐derived reference mitogenomes were updated according to Harbach and Wilkerson (2023), while GenBank accession numbers were retained unchanged to preserve sequence traceability.

For phylogenetic reconstruction, the nucleotide sequences of the 13 mitochondrial protein‐coding genes (PCGs; *ND2*, *COX1*, *COX2*, *ATP8*, *ATP6*, *COX3*, *ND3*, *ND5*, *ND4*, *ND4L*, *ND6*, *CYTB*, and *ND1*) and the two ribosomal RNA genes (16S *rRNA* and 12S *rRNA*) were extracted separately from each mitogenome. Each locus was aligned independently using MAFFT v7 (Katoh and Standley 2013). The resulting 15 locus‐specific alignments were then concatenated into a single supermatrix, while retaining each gene as a separate partition for downstream model selection and phylogenetic inference. This partitioned multigene strategy is consistent with current mitogenomic approaches used to infer evolutionary relationships within Culicidae and related mosquito lineages (Luo et al. 2016; Yurchenko et al. 2020).

To account for heterogeneity in sequence evolution among loci, the best‐fitting nucleotide substitution model for each partition was selected independently using ModelFinder (Kalyaanamoorthy et al. 2017) as implemented in IQ‐TREE 2 (Minh et al. 2020). The final maximum‐likelihood (ML) tree search was then conducted in RAxML‐NG (Kozlov et al. 2019) under a partitioned framework using the concatenated 15‐partition dataset. Nodal support was assessed with 1000 bootstrap replicates. The resulting topology was rooted using an outgroup set comprising three *Cx. coronator* mitogenomes (MF040162.1, MF381691.1, and MF509890.1) together with *Cx. gelidus* (KX753344.1), whereas *Cx. australicus* was retained within the ingroup. The final tree was prepared for figure generation, with the Turkish Black Sea isolates highlighted separately from the remaining taxa.

Because mitochondrial genomes are maternally inherited and previous studies have shown that mitochondrial phylogenies within the *Cx. pipiens* complex may reflect maternal lineage history, introgression, incomplete lineage sorting, and possibly endosymbiont‐associated mitochondrial sweeps rather than strict species‐ or form‐level boundaries, the inferred relationships were interpreted with appropriate caution (Luo et al. 2016; Yurchenko et al. 2020; Aardema et al. 2022). Within this framework, the phylogeny was used to evaluate the maternal evolutionary placement of the Turkish Black Sea isolates relative to previously published mitogenomes and to assess their affinities within the broader *Cx. pipiens* complex.

## 3 Results

### 3.1 General Features of the Mitochondrial Genomes of Turkish *Culex pipiens* Form Pipiens Isolates

Raw paired‐end shotgun reads from all five Turkish isolates produced continuous mitochondrial coverage profiles when remapped to the common *Culex pipiens* reference mitogenome NC_015079. Although local fluctuations in read depth were observed, coverage extended across the full length of the reference genome in every isolate (Figure S1). These local depth differences likely reflect a combination of shotgun‐library coverage heterogeneity, mapping behavior across A + T‐rich or repetitive regions, and possible effects of mitochondrial‐like nuclear sequences. However, final consensus sequences were not based solely on common‐reference mapping; they were confirmed through concordance between reference‐guided and de novo organelle assemblies, reducing the likelihood that local mapping‐depth fluctuations affected the final mitogenome sequences. Corresponding mapping and coverage statistics are summarized in Table S1. Together, these results support the completeness and reliability of the mitochondrial genome assemblies used in downstream comparative analyses.

**FIGURE 2:**
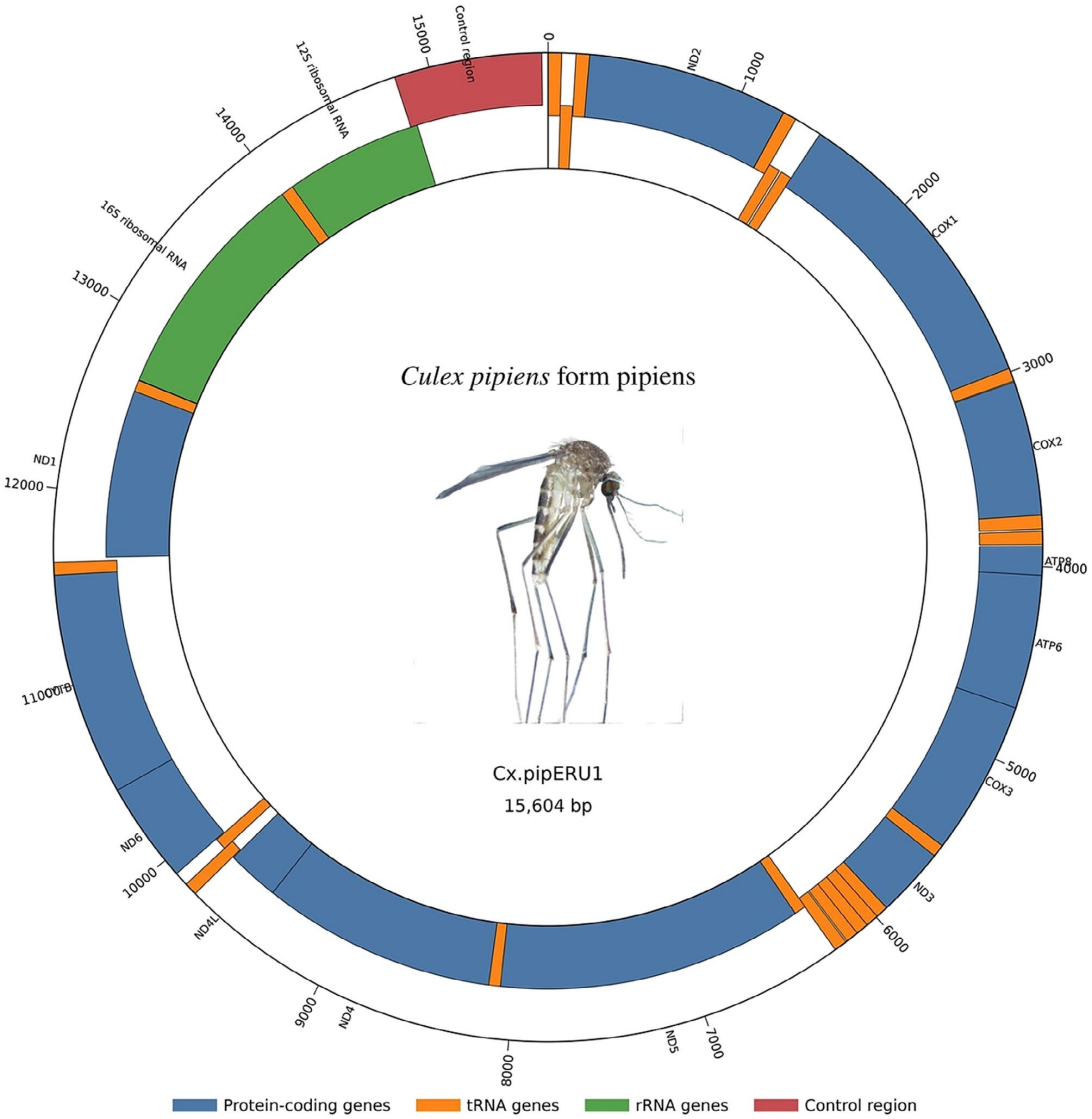
Circular map of the mitochondrial genome of the representative Turkish isolate *Cx. pipiens* form pipiens Cx.pipERU1 (15,604 bp). Protein‐coding genes are shown in blue, tRNA genes in orange, rRNA genes in green, and the control region in red. Genes on opposite strands are displayed on separate radial levels.

The complete mitochondrial genomes of the five Turkish *Cx. pipiens* form pipiens isolates (GenBank accessions: PZ220808–PZ220812) ranged from 15,602 to 15,604 bp in length (Table 2). All mitogenomes contained the typical set of 37 mitochondrial genes, comprising 13 protein‐coding genes (PCGs), 22 transfer RNA (tRNA) genes, and 2 ribosomal RNA (rRNA) genes, together with a control region. A representative circular map of the mitogenome is shown in Figure 2.

**TABLE 2:** General features of the mitochondrial genomes of Turkish *Culex pipiens* form pipiens isolates.

Isolate	Genome length (bp)	A + T (%)	G + C (%)	No. of PCGs	No. of tRNAs	No. of rRNAs	Control region length (bp)	Total PCG length (bp)	Total tRNA length (bp)	Total rRNA length (bp)
Cx.pipERU1	15,604	78.19	21.81	13	22	2	747	11,216	1476	2118
Cx.pipERU2	15,602	78.16	21.84	13	22	2	746	11,216	1475	2118
Cx.pipERU3	15,604	78.19	21.81	13	22	2	747	11,216	1476	2118
Cx.pipERU4	15,603	78.19	21.81	13	22	2	747	11,216	1475	2118
Cx.pipERU5	15,603	78.19	21.81	13	22	2	747	11,216	1475	2118

Overall mitogenome organization was highly conserved among the Turkish isolates. All five assemblies exhibited the same gene complement and gene order, with no evidence of gene rearrangement (Table 3). As in other culicid mitogenomes, genes were distributed across both strands. The control region length showed only slight variation, measuring 746 bp in Cx.pipERU2 and 747 bp in the remaining isolates (Table 2). Total PCG length was identical among all isolates, whereas only minor differences were observed in the combined lengths of tRNA genes and rRNA regions. The total rRNA length was identical in all five Turkish isolates (2118 bp) (Table 2).

**TABLE 3:** Annotated mitochondrial genes of the representative Turkish isolate *Culex pipiens* form *pipiens* Cx.pipERU1 (GenBank accession: PZ220808).

Feature type	Gene	Start	End	Strand	Length (bp)	Start/stop codon
tRNA	tRNA‐Ile	2	69	H	68	—
tRNA	tRNA‐Gln	70	138	L	69	—
tRNA	tRNA‐Met	142	210	H	69	—
CDS	ND2	211	1233	H	1023	ATC/TAA
tRNA	tRNA‐Trp	1235	1303	H	69	—
tRNA	tRNA‐Cys	1304	1369	L	66	—
tRNA	tRNA‐Tyr	1382	1447	L	66	—
CDS	COX1	1446	2982	H	1537	TCG/T
tRNA	tRNA‐Leu	2983	3049	H	67	—
CDS	COX2	3055	3739	H	685	ATG/T
tRNA	tRNA‐Lys	3740	3810	H	71	—
tRNA	tRNA‐Asp	3821	3888	H	68	—
CDS	ATP8	3898	4050	H	153	ATA/TAA
CDS	ATP6	4044	4724	H	681	ATG/TAA
CDS	COX3	4724	5511	H	788	ATG/TA
tRNA	tRNA‐Gly	5512	5578	H	67	—
CDS	ND3	5579	5930	H	352	ATT/T
tRNA	tRNA‐Arg	5931	5994	H	64	—
tRNA	tRNA‐Ala	5995	6060	H	66	—
tRNA	tRNA‐Asn	6061	6127	H	67	—
tRNA	tRNA‐Ser	6126	6190	H	65	—
tRNA	tRNA‐Glu	6198	6263	H	66	—
tRNA	tRNA‐Phe	6262	6328	L	67	—
CDS	ND5	6329	8071	L	1743	GTG/TAA
tRNA	tRNA‐His	8072	8137	L	66	—
CDS	ND4	8138	9480	L	1343	ATG/TA
CDS	ND4L	9474	9770	L	297	ATG/TAA
tRNA	tRNA‐Thr	9776	9841	H	66	—
tRNA	tRNA‐Pro	9842	9907	L	66	—
CDS	ND6	9913	10,428	H	516	ATA/TAA
CDS	CYTB	10,428	11,562	H	1135	ATG/T
tRNA	tRNA‐Ser	11,563	11,628	H	66	—
CDS	ND1	11,647	12,597	L	951	TTG/TAA
tRNA	tRNA‐Leu	12,600	12,664	L	65	—
rRNA	16S ribosomal RNA	12,668	14,000	L	1333	—
tRNA	tRNA‐Val	14,001	14,072	L	72	—
rRNA	12S ribosomal RNA	14,073	14,857	L	785	—

Gene‐by‐gene annotation confirmed the presence of all expected mitochondrial coding genes and structural RNA genes in each isolate, further supporting the completeness of the assembled consensus sequences (Table 3). The representative mitogenome map of Cx.pipERU1 illustrates the conserved structural organization of the Turkish mitogenomes, including the distribution of protein‐coding genes, tRNA genes, and rRNA genes on opposite strands (Figure 2).

### 3.2 Nucleotide Composition and Compositional Skew of the Mitogenomes

The mitochondrial genomes of the five Turkish *Cx. pipiens* form pipiens isolates exhibited highly similar nucleotide composition profiles (Table 4). The overall A + T content of the complete mitogenomes ranged from 78.16% to 78.19%, whereas G + C content varied only slightly between 21.81% and 21.84%, indicating a pronounced AT bias typical of insect mitochondrial genomes.

**TABLE 4:** Nucleotide composition and compositional skew of whole mitogenomes and major genomic components in Turkish *Culex pipiens* form *pipiens* isolates.

Isolate	Region	A	T	G	C	A + T (%)	G + C (%)	AT‐skew	GC‐skew
Cx.pipERU1	Whole mitogenome	6151	6050	1431	1972	78.19	21.81	0.0083	−0.1590
Cx.pipERU1	PCGs	3613	4974	1394	1235	76.56	23.44	−0.1585	0.0605
Cx.pipERU1	tRNAs	589	577	174	136	79.00	21.00	0.0103	0.1226
Cx.pipERU1	rRNAs	848	893	242	135	82.20	17.80	−0.0258	0.2838
Cx.pipERU2	Whole mitogenome	6150	6045	1430	1977	78.16	21.84	0.0086	−0.1606
Cx.pipERU2	PCGs	3608	4975	1397	1236	76.52	23.48	−0.1593	0.0611
Cx.pipERU2	tRNAs	589	576	174	136	78.98	21.02	0.0112	0.1226
Cx.pipERU2	rRNAs	848	895	242	133	82.29	17.71	−0.0270	0.2907
Cx.pipERU3	Whole mitogenome	6154	6047	1427	1976	78.19	21.81	0.0088	−0.1613
Cx.pipERU3	PCGs	3616	4974	1391	1235	76.59	23.41	−0.1581	0.0594
Cx.pipERU3	tRNAs	589	577	174	136	79.00	21.00	0.0103	0.1226
Cx.pipERU3	rRNAs	848	893	242	135	82.20	17.80	−0.0258	0.2838
Cx.pipERU4	Whole mitogenome	6149	6047	1431	1976	78.16	21.84	0.0084	−0.1600
Cx.pipERU4	PCGs	3610	4975	1395	1236	76.54	23.46	−0.1590	0.0604
Cx.pipERU4	tRNAs	589	576	174	136	78.98	21.02	0.0112	0.1226
Cx.pipERU4	rRNAs	848	893	242	135	82.20	17.80	−0.0258	0.2838
Cx.pipERU5	Whole mitogenome	6150	6044	1430	1976	78.17	21.83	0.0087	−0.1603
Cx.pipERU5	PCGs	3611	4975	1395	1234	76.56	23.44	−0.1589	0.0612
Cx.pipERU5	tRNAs	587	576	174	136	78.95	21.05	0.0095	0.1226
Cx.pipERU5	rRNAs	848	893	242	135	82.20	17.80	−0.0258	0.2838

Abbreviations: PCGs, protein‐coding genes; rRNAs, ribosomal RNA genes; tRNAs, transfer RNA genes.

Compositional asymmetry was likewise highly consistent among the five isolates (Table 4). AT‐skew values for the whole mitogenomes were slightly positive, ranging from 0.0083 to 0.0088, indicating a small excess of adenine over thymine. In contrast, GC‐skew values were consistently negative, ranging from −0.1590 to −0.1613, reflecting a clear excess of cytosine over guanine. Together, these results show that the Turkish mitogenomes share a highly conserved nucleotide composition and strand‐asymmetry profile.

**FIGURE 3:**
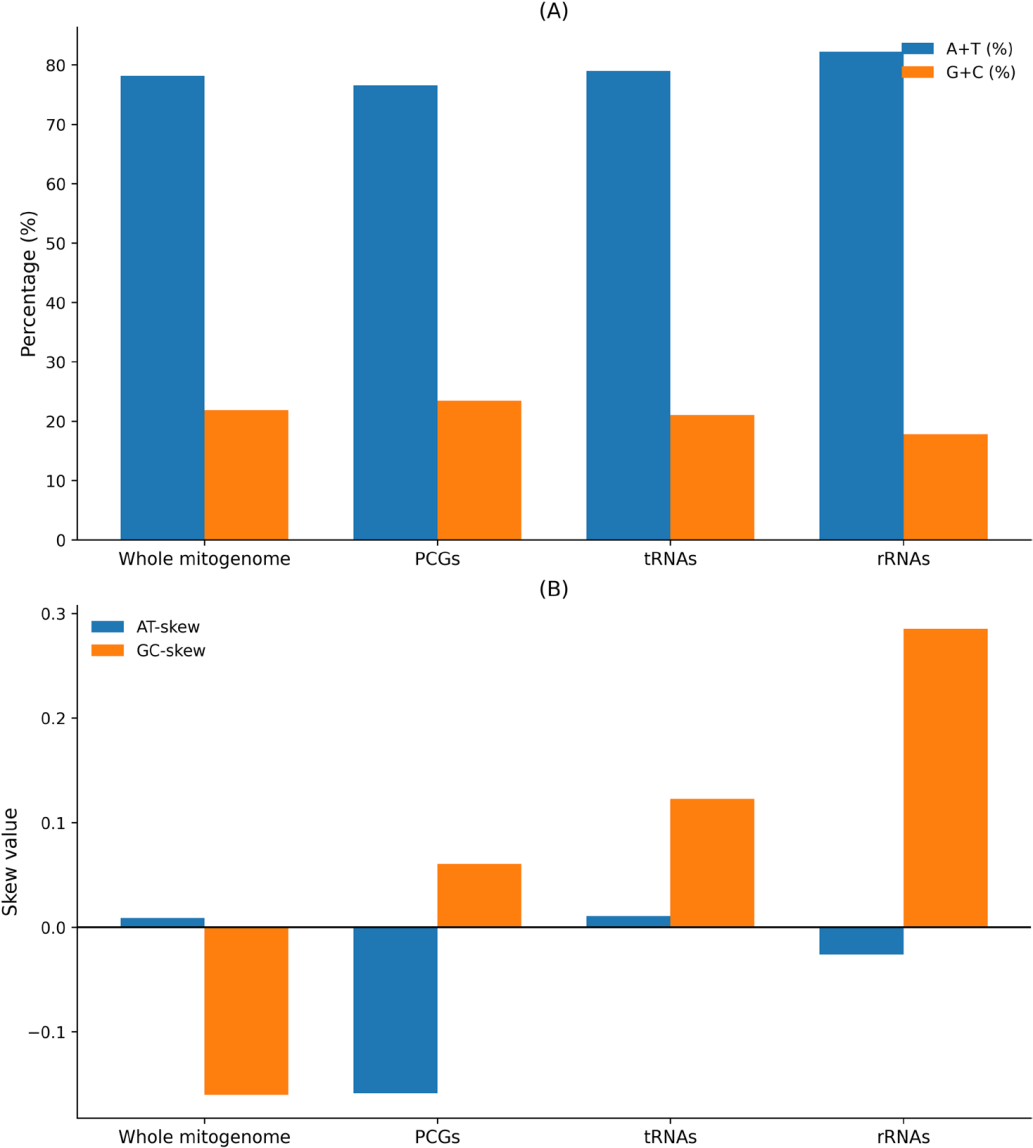
Nucleotide composition and compositional skew of whole mitochondrial genomes and major genomic components in Turkish *Cx. pipiens* form pipiens isolates. (A) Mean nucleotide composition of whole mitogenomes and major genomic components, showing A + T and G + C percentages for complete mitogenomes, protein‐coding genes, tRNA genes, and rRNA genes. (B) Mean compositional skew of whole mitogenomes and major genomic components, showing AT‐skew and GC‐skew values for complete mitogenomes, protein‐coding genes, tRNA genes, and rRNA genes.

When major genomic components were analyzed separately, protein‐coding genes, tRNA genes, and rRNA genes all retained the general AT‐rich pattern observed in the complete mitogenomes (Table 4, Figure 3A). However, slight differences were detected among these genomic partitions in both nucleotide composition and skew values. These patterns are summarized in Figure 3A,B, which show the mean nucleotide composition and compositional skew of the whole mitogenomes and their major genomic components. Overall, the five Turkish isolates displayed a highly uniform compositional profile across both the complete mitogenome and its constituent regions.

### 3.3 Protein‐Coding Genes, Codon Usage, and Amino Acid Composition

**FIGURE 4:**
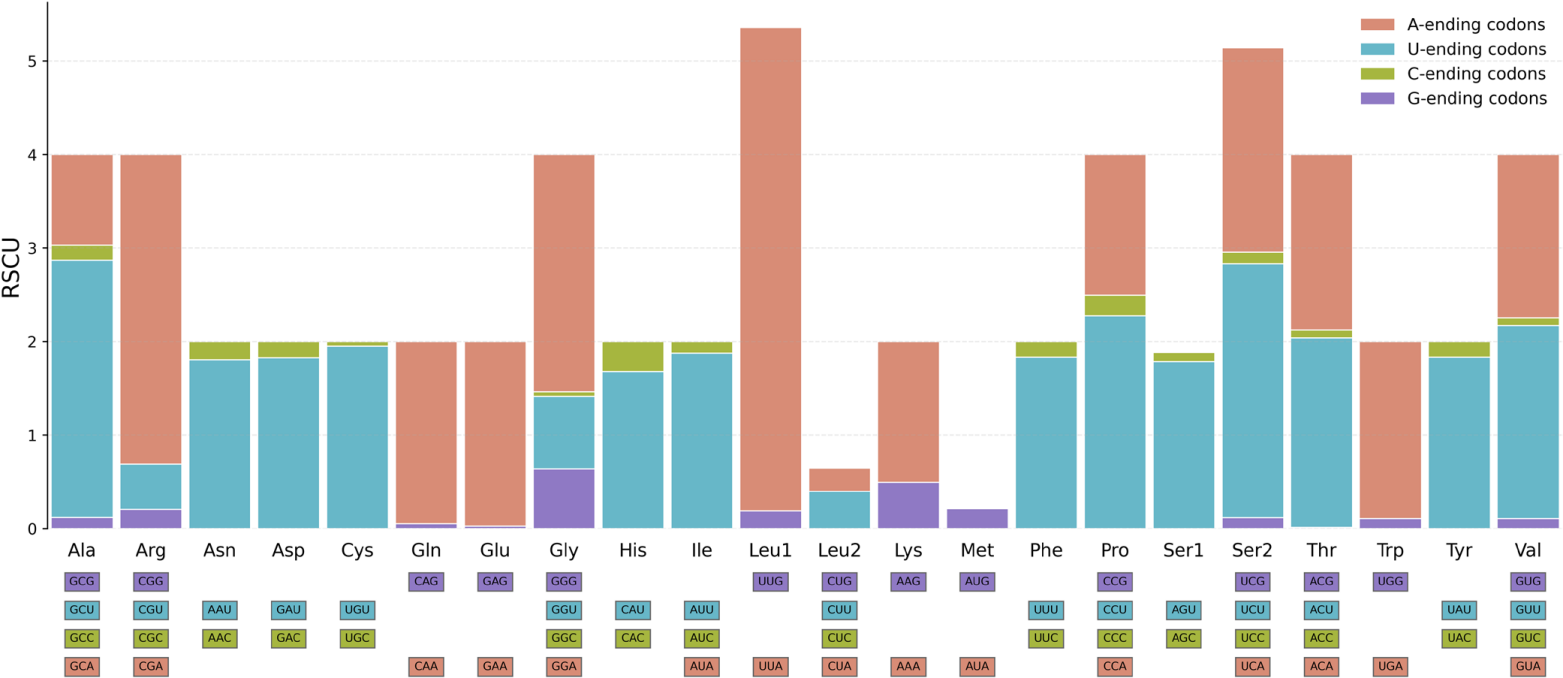
Relative synonymous codon usage (RSCU) of mitochondrial protein‐coding genes in Turkish *Cx. pipiens* form pipiens. Each amino acid is represented by a stacked bar composed of its synonymous codons. Codon segments and codon labels are arranged in a fixed order and colored according to the identity of the third nucleotide position.

The 13 mitochondrial protein‐coding genes (PCGs) of the Turkish *Cx. pipiens* form pipiens isolates exhibited highly similar codon usage patterns, consistent with the overall A + T‐rich character of the mitogenomes. Relative synonymous codon usage (RSCU) analysis showed a clear preference for several A‐ or U‐ending synonymous codons, whereas many C‐ or G‐ending alternatives were comparatively underrepresented (Figure 4). Among the most frequently preferred codons were GCU (Ala), UCU (Ser), CCU (Pro), ACU (Thr), and CGA (Arg), all of which showed relatively high mean RSCU values. In contrast, several synonymous codons, including CGC, AGG, CCG, CUC, and CUG, were absent or occurred only at very low frequencies, indicating pronounced codon usage bias in the Turkish mitogenomes.

The overall RSCU profile was highly conserved among the five Turkish isolates, with only minor quantitative differences. For many amino acid families, codon usage was dominated by one or two synonymous codons, whereas the remaining alternatives contributed only weakly to the overall pattern (Figure 4). These results indicate that codon usage in the Turkish *Cx. pipiens* form pipiens mitogenomes is strongly shaped by nucleotide compositional bias and conserved mitochondrial coding preferences.

**FIGURE 5:**
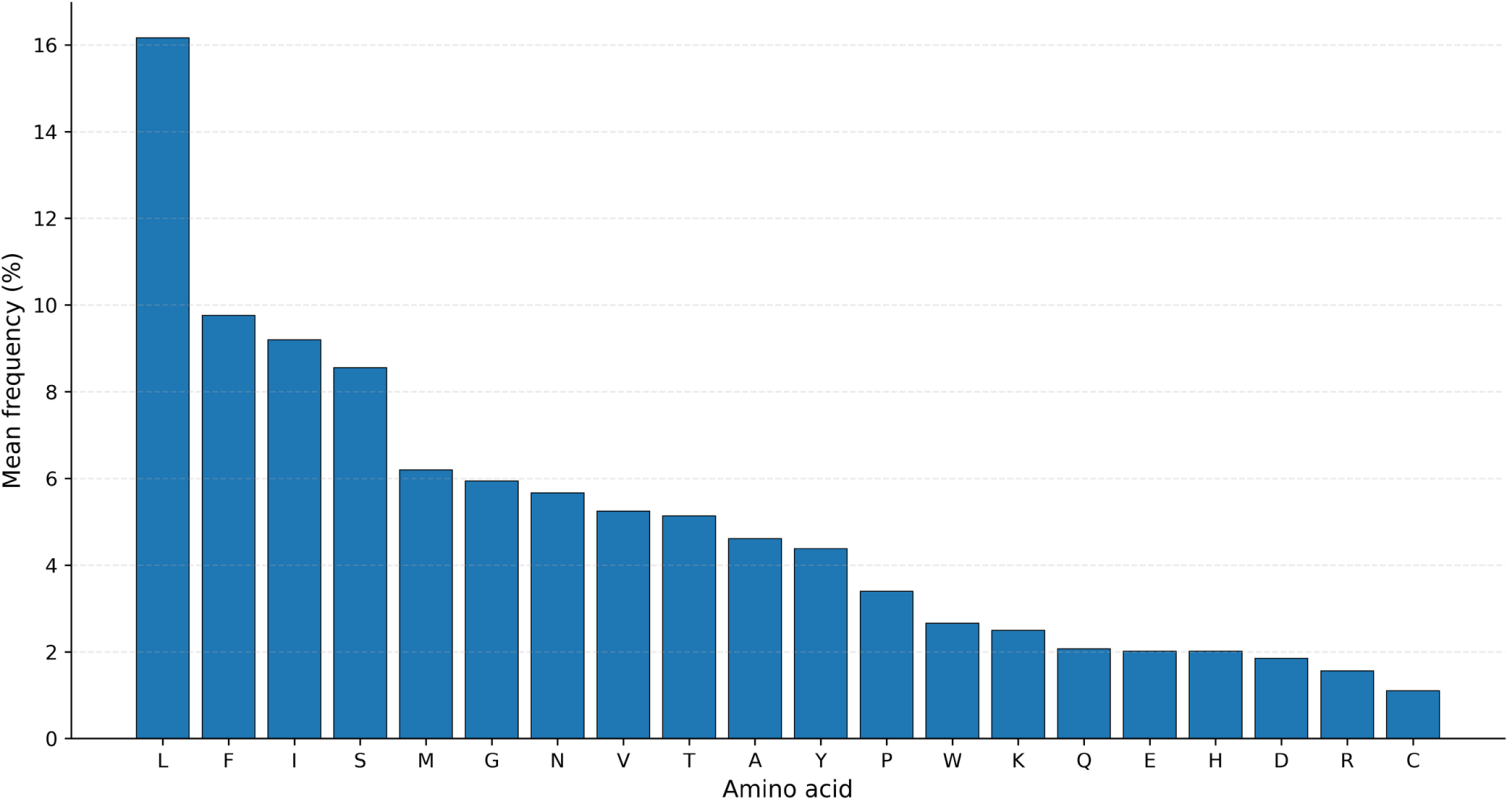
Mean amino acid composition of the concatenated mitochondrial protein‐coding genes in Turkish *Cx. pipiens* form pipiens. Bars represent mean amino acid frequencies calculated across the five Turkish isolates.

The inferred amino acid composition of the concatenated mitochondrial PCGs was likewise highly conserved across all Turkish isolates (Figure 5). Leucine was the most abundant amino acid, followed by phenylalanine, isoleucine, serine, and methionine. By contrast, several other amino acids occurred at relatively low frequencies across the concatenated coding sequences. Overall, the amino acid composition profile was highly homogeneous among the five isolates and showed only minimal variation. Unlike RSCU, which reflects synonymous codon preference, amino acid frequencies more likely reflect the conserved structural and functional constraints of mitochondrial protein‐coding genes.

These compositional and coding‐profile analyses were used to document the baseline features of the newly generated Turkish mitogenomes and to assess their consistency with mitochondrial patterns previously reported for members of the *Cx. pipiens* complex, rather than to imply a distinct Türkiye‐specific compositional signature.

### 3.4 Mitochondrial Sequence Variation Within Turkish Isolates and Across the Broader *Culex pipiens* Complex

Pairwise comparison of the five newly generated Turkish *Cx. pipiens* form pipiens mitogenomes showed low sequence divergence among isolates. Across the complete mitogenomes, p‐distances ranged from 0.000513 to 0.001859, with pairwise differences comprising 8–29 SNPs and 0–2 indel positions (Table 5). The greatest divergence was observed between Cx.pipERU1 and Cx.pipERU2, whereas the lowest divergence was found between Cx.pipERU2 and Cx.pipERU4. These results indicate that the Turkish isolates are highly similar at the mitochondrial genome level, despite being collected from geographically separated localities along the Black Sea region.

**TABLE 5:** Pairwise sequence variation among Turkish *Culex pipiens* form pipiens mitogenomes.

Sequence 1	Sequence 2	Comparable sites	SNPs	Indels	*p*‐distance	Identity (%)
Cx.pipERU1	Cx.pipERU2	15,602	29	2	0.001859	99.8141
Cx.pipERU1	Cx.pipERU3	15,604	13	0	0.000833	99.9167
Cx.pipERU1	Cx.pipERU4	15,603	23	1	0.001474	99.8526
Cx.pipERU1	Cx.pipERU5	15,603	21	1	0.001346	99.8654
Cx.pipERU2	Cx.pipERU3	15,602	22	2	0.001410	99.8590
Cx.pipERU2	Cx.pipERU4	15,602	8	1	0.000513	99.9487
Cx.pipERU2	Cx.pipERU5	15,602	15	1	0.000961	99.9039
Cx.pipERU3	Cx.pipERU4	15,603	16	1	0.001025	99.8975
Cx.pipERU3	Cx.pipERU5	15,603	14	1	0.000897	99.9103
Cx.pipERU4	Cx.pipERU5	15,603	10	0	0.000641	99.9359

**FIGURE 6:**
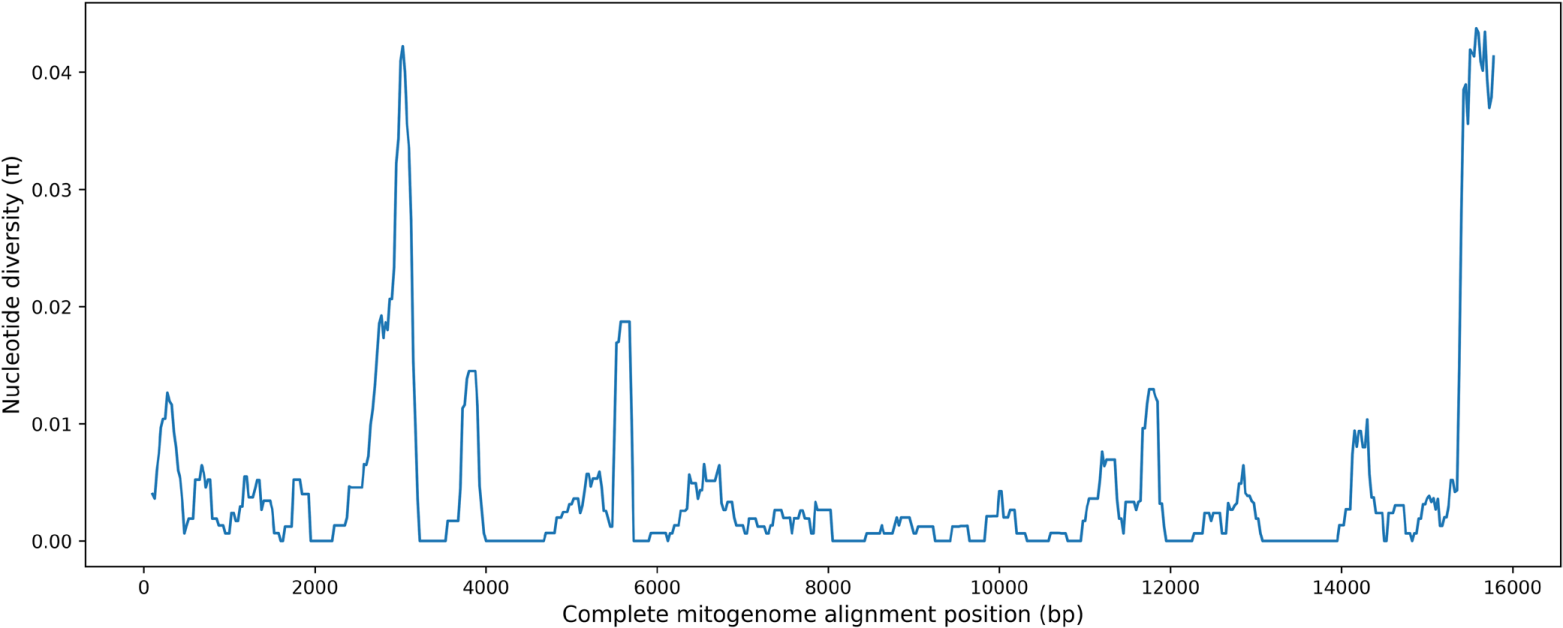
Sliding‐window nucleotide diversity across the broader *Cx. pipiens* complex complete mitogenome dataset. The analysis included the five Turkish *Cx. pipiens* form pipiens mitogenomes together with representative GenBank‐derived mitogenomes of *Cx. pipiens* form pipiens, *Cx. pipiens* form molestus, *Cx. pallens*, and *Cx. quinquefasciatus*. Nucleotide diversity (*π*) was calculated using a 200‐bp sliding window with a 25‐bp step size.

Because the Turkish isolates were not recovered as a single exclusive mitochondrial lineage in the phylogenetic analysis, sequence variation was further evaluated in the context of the broader *Cx. pipiens* complex mitogenome dataset. This expanded dataset included the five Turkish *Cx. pipiens* form pipiens mitogenomes together with representative GenBank‐derived mitogenomes assigned to *Cx. pipiens* form pipiens, *Cx. pipiens* form molestus, *Cx. pallens*, and *Cx. quinquefasciatus*. Sliding‐window analysis of the complete mitogenome alignment showed that nucleotide diversity was unevenly distributed across the broader complex dataset (Figure 6, Table S2). Most of the mitogenome exhibited low sequence diversity, whereas several localized regions showed elevated *π* values. The most pronounced diversity peaks occurred in the central portion of the alignment, approximately around 2.8–3.2 kb, and near the terminal portion of the mitogenome, approximately around 15.4–15.8 kb. The terminal peak is consistent with the expected higher variability of the A + T‐rich control region and adjacent non‐coding segments. Additional moderate peaks were observed in several internal regions of the mitogenome, indicating that sequence variation across the complex is concentrated in discrete mitochondrial intervals rather than being evenly distributed across the genome.

**FIGURE 7:**
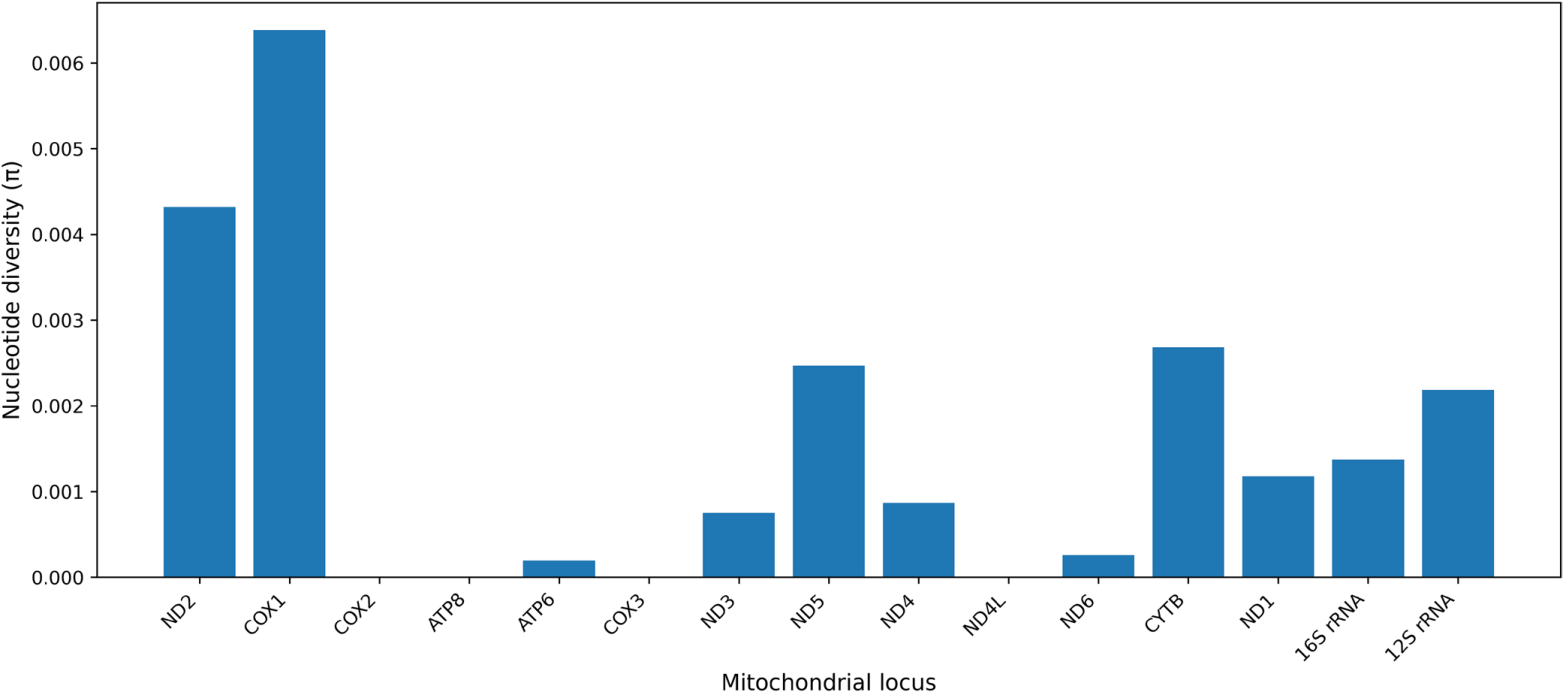
Gene‐based nucleotide diversity across the broader *Cx. pipiens* complex dataset. Nucleotide diversity (*π*) was calculated separately for the 13 mitochondrial protein‐coding genes and two rRNA genes using the expanded ingroup dataset including the Turkish isolates and representative GenBank‐derived mitogenomes.

Gene‐based nucleotide diversity analysis of the broader dataset further showed that the contribution of individual mitochondrial loci to overall variation was highly uneven (Figure 7, Table S3). Among the 13 protein‐coding genes and two rRNA genes, COX1 exhibited the highest nucleotide diversity (*π* = 0.006382), followed by ND2 (*π* = 0.004320), COX3 (*π* = 0.002900), CYTB (*π* = 0.002684), ND5 (*π* = 0.002467), and 12S rRNA (*π* = 0.002183). In contrast, ATP8 and ND4L showed no detectable nucleotide diversity in this dataset, while ATP6 and ND6 were among the most conserved loci. Overall, the broader complex‐level analysis showed that the Turkish isolates fall within the general mitochondrial diversity of the *Cx. pipiens* complex rather than representing a distinct or highly divergent regional mitochondrial profile. Thus, their main contribution is to expand the geographic representation of complete mitogenomes from the Turkish Black Sea region and to improve regional sampling of maternal lineages within the broader complex.

### 3.5 Structural Features of tRNA and rRNA Genes

The mitochondrial genomes of the Turkish *Cx. pipiens* form pipiens isolates contained the standard complement of 22 transfer RNA (tRNA) genes and two ribosomal RNA (rRNA) genes, namely 16S rRNA and 12S rRNA. The overall organization of these structural RNA genes was highly conserved among the five Turkish isolates, indicating strong conservation of the non‐coding RNA fraction of the mitogenome.

Secondary structure predictions for the representative isolate Cx.pipERU1 showed recognizable folded structures for all 22 mitochondrial tRNAs (Figure S2). The inferred tRNA set included the expected two leucine tRNAs, tRNA‐Leu (UUR) and tRNA‐Leu (CUN), as well as the two serine tRNAs, tRNA‐Ser (AGN) and tRNA‐Ser (UCN), all of which were recovered in the final structural panel. Overall, the predicted tRNA structures were consistent with the typical organization of insect mitochondrial tRNAs.

Secondary structure plots were also obtained for the two mitochondrial ribosomal RNA genes (Figure S3). These analyses showed that both 16S rRNA and 12S rRNA formed complex folded structures, consistent with their roles as the large and small ribosomal RNA subunits, respectively. In the representative Turkish mitogenome, the ribosomal RNA region retained the conserved arrangement in which rrnL is followed by trnV and then rrnS.

Sequence variation in the structural RNA genes was generally limited among the Turkish isolates. Most tRNA genes were completely conserved, and only minimal variation was detected within this fraction. Among the two ribosomal RNA genes, 12S rRNA showed low but detectable variability (Pi = 0.001019), whereas 16S rRNA remained fully conserved in the final alignment (Table S2). Overall, these results indicate that the structural RNA fraction of the Turkish *Cx. pipiens* form pipiens mitogenomes is highly conserved at both the sequence and predicted secondary‐structure levels.

### 3.6 Control Region Analysis

The mitochondrial control region was highly conserved among the five Turkish *Cx. pipiens* form pipiens isolates, with only slight length variation across the dataset. Consistent with the overall compositional bias of the mitogenome, this region was strongly A + T‐rich in all isolates. A comparative summary of control‐region length, A + T content, dominant short repeat candidates, and representative stem‐loop potential is provided in Figure S4.

Structural examination of the control region identified short TT‐dominated repeat motifs and a conserved stem‐loop potential across the Turkish isolates. These observations indicate that, despite limited overall size variation, the control region retains the characteristic combination of compositional bias and localized structural complexity typical of mosquito mitogenomes.

Although the control region itself was structurally conserved, the genomic intervals adjacent to it showed some of the highest nucleotide diversity values detected across the mitogenome. Together with the sliding‐window results presented above, this pattern suggests that informative sequence variation in the Turkish isolates is concentrated not only in selected protein‐coding genes but also in regions flanking the control region. These regions may therefore provide useful targets for future fine‐scale population genetic and phylogeographic analyses within *Cx. pipiens* form pipiens.

### 3.7 Phylogenetic Relationships Within the *Culex pipiens* Complex

**FIGURE 8:**
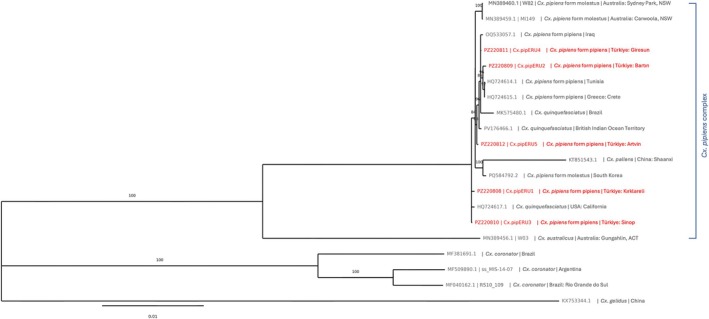
Partitioned maximum‐likelihood phylogeny of the *Cx. pipiens* complex based on concatenated mitochondrial sequences of 13 protein‐coding genes and 2 rRNA genes. The analysis was conducted in RAxML‐NG with 1000 bootstrap replicates. The tree was rooted with *Cx. coronator* (MF040162.1, MF381691.1, MF509890.1) and *Cx. gelidus* (KX753344.1); *Cx. australicus* was retained within the ingroup. Taxon labels were updated according to Harbach and Wilkerson (2023), while GenBank accession numbers were retained unchanged. Turkish Black Sea isolates are shown in red. Branch lengths indicate substitutions per site.

Phylogenetic relationships among the analyzed mitogenomes were inferred from the concatenated nucleotide sequences of 13 mitochondrial protein‐coding genes and two mitochondrial rRNA genes under a partitioned maximum‐likelihood framework (Figure 8). The final strict matrix comprised 20 complete mitogenomes, including the five newly generated Turkish *Cx. pipiens* form pipiens isolates, representative ingroup mitogenomes of *Cx. pipiens* form pipiens, *Cx. pipiens* form molestus, *Cx. pallens*, *Cx. quinquefasciatus*, and *Cx. australicus*, and an outgroup set consisting of three *Cx. coronator* mitogenomes together with *Cx. gelidus*. Nodal support was assessed with 1000 bootstrap replicates.

The outgroup taxa formed a clearly separated basal assemblage. Within this external group, the three *Cx. coronator* mitogenomes clustered together, with MF040162.1 and MF509890.1 recovered as a strongly supported pair, whereas *Cx. gelidus* occupied the most distant external position. In contrast, *Cx. australicus* was retained within the broader ingroup framework and represented the deepest ingroup lineage in the present dataset. Overall, the topology resolved members of the *Cx. pipiens* complex into several closely related but partially intermixed maternal lineages rather than into fully discrete groups corresponding to species or form designations.

Within the principal *Cx. pipiens* complex assemblage, the two Australian *Cx. pipiens* form molestus mitogenomes (MN389459.1 and MN389460.1) formed a strongly supported pair. However, the remaining lineages were not partitioned into reciprocally monophyletic clusters corresponding to *Cx. pipiens* form pipiens, *Cx. pipiens* form molestus, *Cx. pallens*, or *Cx. quinquefasciatus*. Notably, KT851543.1 (*Cx. pallens*, China: Shaanxi) and PQ584792.2 (*Cx. pipiens* form molestus, South Korea) also formed a strongly supported pair despite their different species or form designation. Several deeper internal nodes within the complex showed only moderate or variable support.

The Turkish *Cx. pipiens* form pipiens isolates were distributed across multiple parts of the ingroup topology rather than forming a single exclusive clade. PZ220811 (Cx.pipERU4) and PZ220809 (Cx.pipERU2) occurred within the same upper sector of the tree together with reference *Cx. pipiens* form pipiens mitogenomes from Tunisia, Greece, and Iraq. This broader assemblage also included *Cx. quinquefasciatus* mitogenomes from Brazil and the British Indian Ocean Territory, as well as the Turkish isolate PZ220812 (Cx.pipERU5). In another part of the tree, PZ220808 (Cx.pipERU1) clustered near the *Cx. quinquefasciatus* mitogenome from the USA, while PZ220810 (Cx.pipERU3) branched in close proximity to that pair. Thus, the five Turkish mitogenomes were recovered in at least two distinct sectors of the ingroup rather than as a unified Turkish mitochondrial lineage.

Taken together, these results show that complete mitochondrial genomes do not recover fully discrete maternal groupings for the recognized members of the *Cx. pipiens* complex in the present dataset. Instead, the inferred topology supports substantial mitochondrial affinity among *Cx. pipiens* form pipiens, *Cx. pipiens* form molestus, *Cx. pallens*, and *Cx. quinquefasciatus*, and places the Turkish Black Sea isolates within this broader pattern of mitogenomic complexity.

## 4 Discussion

The *Cx. pipiens* complex remains one of the most epidemiologically significant and taxonomically challenging mosquito groups worldwide. Accurate characterization of its constituent taxa is critical, as ecological divergence, host‐feeding preferences, and ongoing hybridization within the complex can strongly influence vectorial capacity and, consequently, pathogen transmission dynamics. In the present study, we assembled and conducted a comparative analysis of the complete mitochondrial genomes of five *Cx. pipiens* form pipiens isolates collected from the Turkish Black Sea region. These mitogenomes were highly conserved in size, gene content, and organization, each containing the canonical set of 37 mitochondrial genes typically observed in culicid insects. Their shared architecture, including conserved gene order and strand distribution, further underscores the structural stability of the mitochondrial genome within this complex.

### 4.1 Structural Conservation and Compositional Features of Turkish Mitogenomes

One of the principal findings of this study is the pronounced structural conservation of the Turkish *Culex pipiens* form pipiens mitogenomes. Genome size exhibited only minimal variation across isolates, and all five assemblies retained the same gene complement, gene order, and overall genomic organization. Each mitogenome contained the canonical set of 37 mitochondrial genes typical of culicid insects, while the conserved distribution of genes between the heavy and light strands provides further evidence of the mitochondrial genomic stability within this complex. The structural RNA fraction was similarly conserved, with all 22 tRNA genes and both rRNA genes consistently recovered across isolates and displaying only limited sequence variation.

A pronounced A + T compositional bias was observed across all Turkish isolates, with only minimal variation among genomes. This pattern was also reflected in codon usage, where A‐ or U‐ending synonymous codons were preferentially used. The compositional conservatism observed here is consistent with previous mosquito mitogenome studies, including *Cx. pipiens* form molestus and *Cx. pallens*, in which strong A + T enrichment, similar strand asymmetry, and biased synonymous codon usage were likewise reported. In this respect, the Turkish *Cx. pipiens* form pipiens isolates fit well within the broader compositional framework already described for culicid mitogenomes (Luo et al. 2016).

Another notable feature of the Turkish mitogenomes was the occurrence of non‐canonical initiation and incomplete termination codons in several protein‐coding genes. In the representative annotation, COX1 was inferred to initiate with the unusual start codon TCG, whereas COX1, COX2, COX3, ND3, ND4, and CYTB terminated with incomplete stop codons represented as T or TA. These patterns should not be regarded as anomalous. Luo et al. (2016) explicitly reported TCG as the initiation codon of COX1 in *Culex* and showed that COX1 and COX2 may end with an incomplete terminal T, which is presumed to be completed to TAA through post‐transcriptional polyadenylation. They further noted that termination codons may vary among *Culex* mitogenomes in genes such as COX3, CYTB, ND3, and ND4. Likewise, Akintola and Hwang (2026) described incomplete stop codons (T or TA) as a common feature of mosquito mitogenomes and more broadly of insect mitochondrial genomes. Accordingly, the codon configuration observed in the Turkish isolates is best interpreted as a normal component of culicid mitochondrial gene expression and annotation, rather than as a sequencing or annotation artifact (Luo et al. 2016; Akintola and Hwang 2026).

### 4.2 Comparative Variation and Informative Mitochondrial Regions

Although pairwise divergence among the five Turkish *Cx. pipiens* form pipiens isolates was low, the expanded comparative analysis across the broader *Cx. pipiens* complex showed that mitochondrial sequence variation is not evenly distributed across the genome. This broader analytical framework was important because the Turkish isolates were not recovered as a single exclusive mitochondrial lineage in the phylogenetic analysis. Therefore, their sequence variation is best interpreted within the wider maternal‐lineage diversity of the complex rather than only as a geographically restricted within‐Türkiye pattern.

In the expanded dataset, gene‐based nucleotide diversity was highest in COX1 and ND2, followed by COX3, CYTB, ND5, and 12S rRNA. This broader pattern differs from the narrower within‐Türkiye comparison, in which only a small number of variable sites were present among the five newly generated mitogenomes. Accordingly, the complex‐level analysis provides a more informative framework for identifying mitochondrial regions that vary across the *Cx. pipiens* complex as a whole. These results also support the view that different mitochondrial loci can differ substantially in their discriminatory value among closely related mosquito taxa, and that reliance on a single short barcode fragment may not capture the full distribution of mitochondrial variation across the mitogenome.

The sliding‐window analysis further showed that nucleotide diversity was concentrated in discrete regions rather than being uniformly distributed across the mitochondrial genome. Pronounced diversity peaks were detected in the central portion of the alignment, approximately around 2.8–3.2 kb, and near the terminal portion of the mitogenome, approximately around 15.4–15.8 kb. The terminal peak is consistent with the expected higher variability of the A + T‐rich control region and adjacent non‐coding segments. This pattern agrees with previous mosquito mitogenome studies showing that the control region is often among the most structurally dynamic and compositionally biased regions of the mitochondrial genome, particularly because of tandem repeat variation and insertion–deletion polymorphisms (Luo et al. 2016; Akintola and Hwang 2026).

The identification of ND2 as one of the more variable loci in the broader dataset is also consistent with recent comparative work suggesting that loci outside the standard COX1 barcode may provide useful discriminatory information in mosquitoes. In a broad survey of European mosquito mitogenomes, van der Beek et al. (2025) reported that ND2 and ND6 can show greater variability than the COX1 barcoding region in some contexts and may therefore be useful for marker development. In the present expanded dataset, ND2 was among the most variable loci, whereas ND6 remained relatively conserved. This difference highlights that the relative informativeness of mitochondrial markers may depend on taxonomic scale, geographic sampling, and the composition of the comparative dataset.

Importantly, the Turkish isolates did not show a unique or highly divergent regional mitochondrial profile when evaluated within the broader *Cx. pipiens* complex dataset. Their main contribution is therefore not the discovery of a structurally distinct Turkish mitochondrial lineage, but the expansion of complete mitogenome representation from the Turkish Black Sea region and the integration of these maternal lineages into a wider comparative framework. These results strengthen the value of the newly generated mitogenomes as regional reference data for future phylogeographic, surveillance‐oriented, and population‐level studies of the *Cx. pipiens* complex.

### 4.3 Maternal Phylogenetic Complexity Within the *Culex pipiens* Complex

The phylogenetic analysis based on the concatenated mitochondrial dataset comprising 13 protein‐coding genes and two rRNA genes further underscores the evolutionary complexity of the *Cx. pipiens* complex. Under the current taxonomic interpretation of Harbach and Wilkerson (2023), *Cx. pallens* is treated as a species and molestus as a physiological form of *Cx. pipiens*. Within this framework, the Turkish *Cx. pipiens* form pipiens isolates did not form a single exclusive mitochondrial lineage but were instead distributed across different parts of the ingroup and associated with *Cx. pipiens* form pipiens, *Cx. pipiens* form molestus, *Cx. pallens*, and *Cx. quinquefasciatus* mitogenomes. This pattern is consistent with the long‐recognized difficulty of resolving the complex into sharply delimited mitochondrial lineages, given that substantial ecological and behavioral differentiation occurs against a background of limited morphological divergence, admixture, and incomplete reproductive isolation (Aardema et al. 2022; Harbach and Wilkerson 2023). This topology supports the expanded comparative framework adopted in the sequence‐variation analyses, because the Turkish mitogenomes are embedded within the wider maternal diversity of the complex rather than representing a discrete regional mitochondrial lineage.

This interpretation is also compatible with the view that the pipiens and molestus forms, although classically distinguished by traits such as diapause, mating behavior, habitat use, autogeny, and host preference, do not represent universally discrete entities across their range. Instead, they appear to form part of a broader ecological and evolutionary mosaic in which local sympatry, intermediacy, and hybridization can occur (Haba and McBride 2022; Haba et al. 2025; Alvial et al. 2024). Within such a framework, the topological mixing observed here between Turkish *Cx. pipiens* form pipiens mitogenomes and *Cx. quinquefasciatus* lineages is biologically plausible. Recent evidence indicates that introgression and hybrid‐zone dynamics in the *Cx. pipiens* complex can be geographically extensive and ecologically contingent, with incomplete reproductive isolation facilitating the movement of alleles and lineage signals across species‐ or form‐level boundaries (Saarman et al. 2025). Earlier genetic studies of *Cx. pipiens* form molestus and *Cx. pipiens* form pipiens also support this interpretation, showing detectable differentiation but reduced rather than absent gene flow, indicating that ecological divergence in this group does not necessarily translate into strict phylogenetic exclusivity (Kent et al. 2007).

The clustering of mitogenomes assigned to *Cx. pipiens* form pipiens, *Cx. pipiens* form molestus, *Cx. pallens*, and *Cx. quinquefasciatus* should therefore not be interpreted as evidence of misidentification of the Turkish specimens. Rather, because mitochondrial genomes are maternally inherited and represent a single non‐recombining genomic compartment, this pattern most likely reflects shared maternal haplotypes, historical introgression, incomplete lineage sorting, and possibly endosymbiont‐associated mitochondrial sweeps. Thus, the mitochondrial tree is best interpreted as a maternal‐lineage hypothesis rather than as a definitive species‐ or form‐delimitation tree for the *Cx. pipiens* complex.

Taken together, our results suggest that mitogenomic data in the *Cx. pipiens* complex are most informative for tracing maternal affinities and historical lineage structure, rather than for defining all species and form boundaries in isolation. Accordingly, complete resolution of relationships within this medically important complex will require integration of mitochondrial data with nuclear markers, explicit tests of admixture, and broader geographic sampling across regions where evolutionary transitions, secondary contact, and ecological divergence are especially informative (Aardema et al. 2022; Haba and McBride 2022; Haba et al. 2025; Alvial et al. 2024; Saarman et al. 2025).

### 4.4 Limitations and Future Directions

This study has several limitations. First, although the five Turkish isolates represent geographically separated localities along the Black Sea coast, the sample size remains limited and therefore cannot capture the full mitochondrial diversity of *Cx. pipiens* populations in Türkiye. Second, mitochondrial genomes are maternally inherited and should not be used alone to delimit species, ecological forms, or hybrid categories within the *Cx. pipiens* complex. Third, differences in genome length among publicly available reference mitogenomes may partly reflect variation in assembly completeness, annotation boundaries, or recovery of the A + T‐rich control region. In addition, the broader comparative analyses were limited by the number and geographic distribution of complete mitogenomes currently available in GenBank, as well as by potential differences in assembly completeness and annotation boundaries among publicly available reference mitogenomes, particularly in the A + T‐rich control region.

Although the sequencing strategy generated total genomic shotgun reads rather than mitochondria‐enriched libraries, the present study was designed as a mitogenome‐focused analysis. We therefore used the whole‐genome read pools only for mitochondrial genome reconstruction and did not attempt nuclear genome‐wide variant calling, population structure analysis, or admixture inference. Such analyses require a separate analytical framework, including genome‐wide read mapping, variant filtering, genotype calling, missing‐data assessment, and explicit evaluation of nuclear population structure. Given the limited sample size of five individuals, nuclear genomic analyses are better suited to a larger population‐level study specifically designed to test admixture and nuclear differentiation within the *Cx. pipiens* complex.

Future work should combine expanded geographic sampling with nuclear genomic markers, explicit admixture analyses, and broader population‐level sampling to clarify the evolutionary and epidemiological structure of the *Cx. pipiens* complex in Türkiye and surrounding regions.

## 5 Conclusion

In conclusion, the complete mitochondrial genomes of five *Culex pipiens* form pipiens isolates from the Turkish Black Sea region revealed a highly conserved genomic architecture, including stable gene content, gene order, nucleotide composition, and structural RNA organization. Despite this overall conservation, expanded comparisons across the broader *Cx. pipiens* complex dataset showed that mitochondrial diversity was concentrated in selected loci, including COX1, ND2, COX3, CYTB, ND5, and 12S rRNA, as well as in terminal regions associated with the A + T‐rich control region. These results highlight potentially informative mitochondrial regions for future comparative, phylogeographic, and population‐level studies.

At the phylogenetic level, the Turkish isolates did not form a single exclusive mitochondrial lineage; instead, they were distributed across different sectors of the broader *Cx. pipiens* complex, together with mitogenomes assigned to *Cx. pipiens* form molestus, *Cx. pallens*, and *Cx. quinquefasciatus*. This pattern supports the interpretation that mitochondrial genomes in this complex primarily reflect maternal lineage history and are influenced by processes such as introgression and incomplete lineage sorting, rather than corresponding to clearly delineated species‐ or form‐level boundaries. Collectively, the mitogenomic data generated in this study provide a valuable regional reference for Turkish populations and enhance current understanding of evolutionary diversity within one of the most epidemiologically significant mosquito vector complexes. Future work integrating nuclear genomic markers, broader geographic sampling, and explicit admixture analyses will be essential for resolving the evolutionary and epidemiological structure of the *Cx. pipiens* complex with greater accuracy.

## Author Contributions

**Ahsen Meliha Toroslu:** data curation (lead), formal analysis (equal), investigation (equal), visualization (equal), writing – original draft (lead). **Alparslan Yildirim:** conceptualization (equal), funding acquisition (lead), project administration (lead), supervision (lead), writing – review and editing (equal). **Gokmen Zafer Pekmezci:** investigation (equal), methodology (equal), resources (equal), writing – original draft (equal). **Osman Ibis:** formal analysis (equal), methodology (equal), writing – original draft (equal). **Samba Deguene Diop:** formal analysis (equal), investigation (equal), methodology (equal), visualization (equal). **Batuhan Askim Arslanhan:** formal analysis (equal), investigation (equal), methodology (equal). **Saffet Teber:** data curation (equal), investigation (equal), methodology (equal). **Alina Denis Kizgin:** formal analysis (equal), investigation (equal), methodology (equal). **Simge Sahin:** investigation (equal), methodology (equal). **Filiz Gunay:** methodology (equal), validation (equal), writing – review and editing (equal). **Nathan Burkett‐Cadena:** conceptualization (equal), supervision (equal), writing – review and editing (equal). **Lindsay Campbell:** methodology (equal), supervision (equal), writing – review and editing (equal). **Abdullah Inci:** writing – review and editing (equal). **Barry Wilmer Alto:** conceptualization (equal), supervision (equal), validation (equal), writing – review and editing (equal).

## Funding

This work was supported by an award from the Defence Threat Reduction Agency (Grant/Award No. HDTRA12210015) for the project “Enhancing arbovirus surveillance and risk management in the public health systems of Georgia, Türkiye, and Ukraine.” The content of the information does not necessarily reflect the position or the policy of the federal government, and no official endorsement should be inferred. Additional support was provided by the Scientific Research Projects Unit of Erciyes University (TYG‐2024‐13666).

## Conflicts of Interest

The authors declare no conflicts of interest.

## Supporting information


**Figure S1:** Mitogenome‐wide read‐depth profiles of the five Turkish *Cx. pipiens* form pipiens isolates.
**Figure S2:** MITOS2‐derived secondary structure plots of the 22 mitochondrial tRNA genes.
**Figure S3:** MITOS2‐derived secondary structure plots of mitochondrial ribosomal RNA genes.
**Figure S4:** Control region features of the Turkish *Cx. pipiens* form pipiens mitogenomes.

**Table S1:** Sequencing output, mitochondrial read mapping, and coverage summary statistics for the five Turkish *Culex pipiens* form pipiens isolates after remapping raw paired‐end reads to the common reference mitogenome NC_015079.
**Table S2:** Feature‐based sequence variation across the broader *Cx. pipiens* complex complete mitogenome dataset.
**Table S3:** Gene‐based nucleotide diversity across the broader *Cx. pipiens* complex dataset.

## Data Availability

The newly generated mitochondrial genome sequences have been deposited in GenBank under accession numbers PZ220808–PZ220812. Appendix tables and appendix figures are provided with the manuscript. Additional data supporting the findings of this study are available from the corresponding author upon reasonable request.
