# Supplementary material for: Mitogenomics of Culex pipiens Form Pipiens From the Turkish Black Sea Region Reveals Structural Conservation and Phylogenetic Complexity
Source: Ecol Evol. 2026 Aug 2;16(8):e74085. doi: 10.1002/ece3.74085 (PMC13429355; doi:10.1002/ece3.74085)
Supplement: Supplementary file 1 — Figure S1: Mitogenome‐wide read‐depth profiles of the five Turkish Cx. pipiens form pipiens isolates. Figure S2: MITOS2‐derived secondary structure plots of the 22 mitochondrial tRNA genes. Figure S3: MITOS2‐derived secondary structure plots of mitochondrial ribosomal RNA genes. Figure S4: Control region features of the Turkish Cx. pipiens form pipiens mitogenomes. [file ECE3-16-e74085-s001.docx]

**Appendix Figures**

**
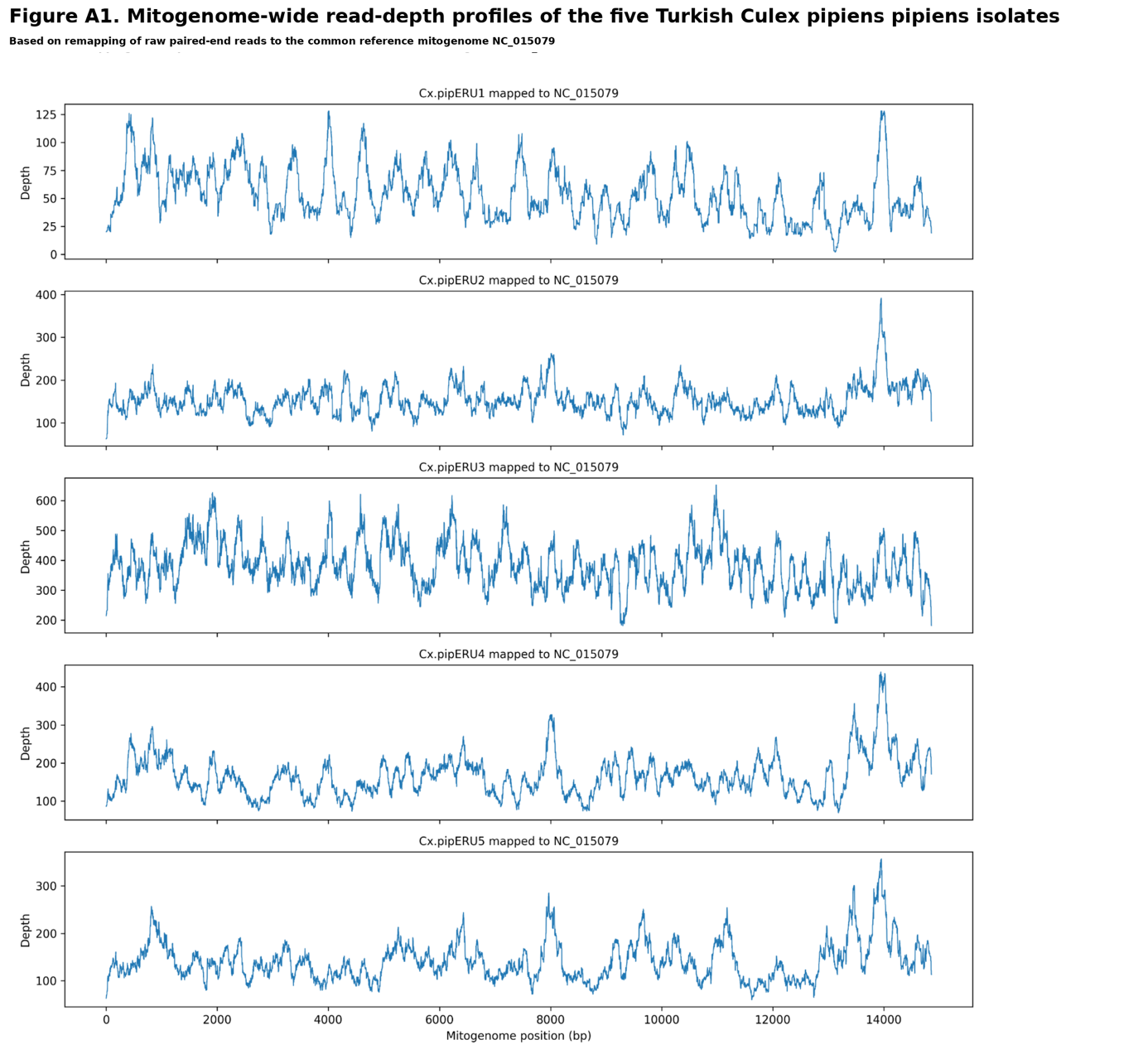
**

**Figure A1.** Mitogenome-wide read-depth profiles of the five Turkish *Culex pipiens* form pipiens isolates. Based on remapping of raw paired-end reads to the common reference mitogenome NC_015079.


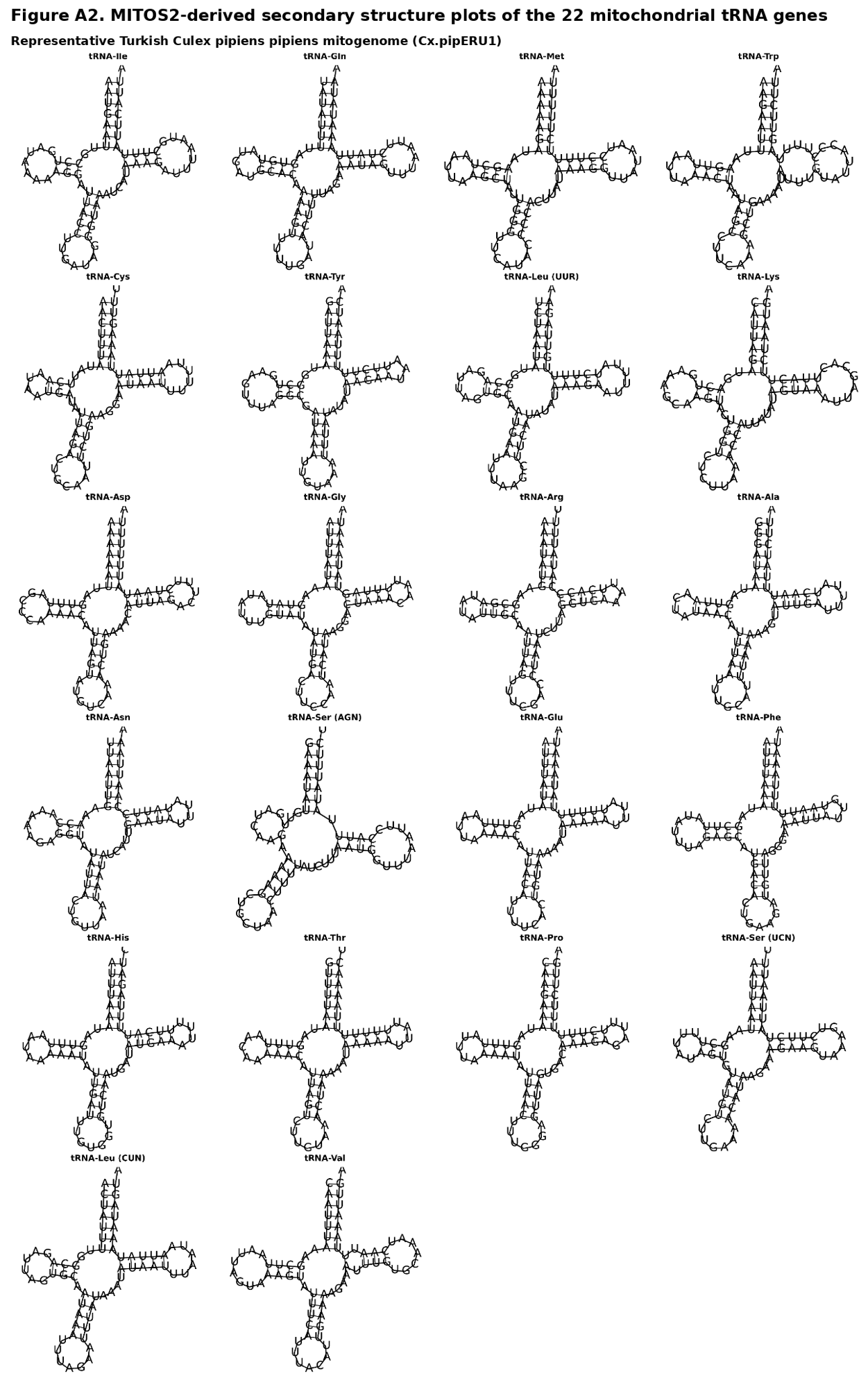


**Figure A2.** MITOS2-derived secondary structure plots of the 22 mitochondrial tRNA genes. Representative Turkish *Culex pipiens* form pipiens mitogenome (Cx.pipERU1).

**
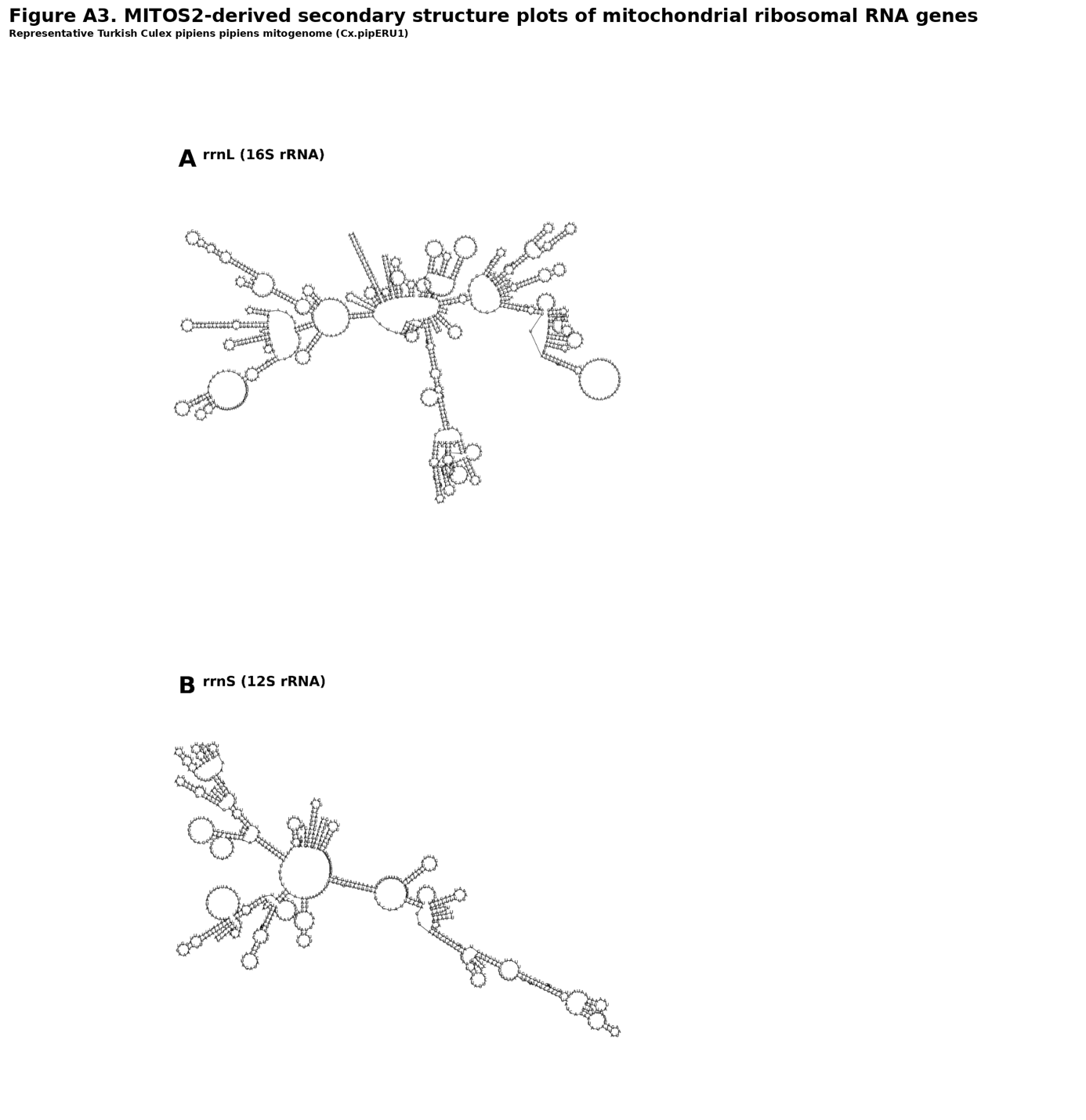
**

**Figure A3.** MITOS2-derived secondary structure plots of mitochondrial ribosomal RNA genes. Representative Turkish *Culex pipiens* form pipiens mitogenome (Cx.pipERU1).

**
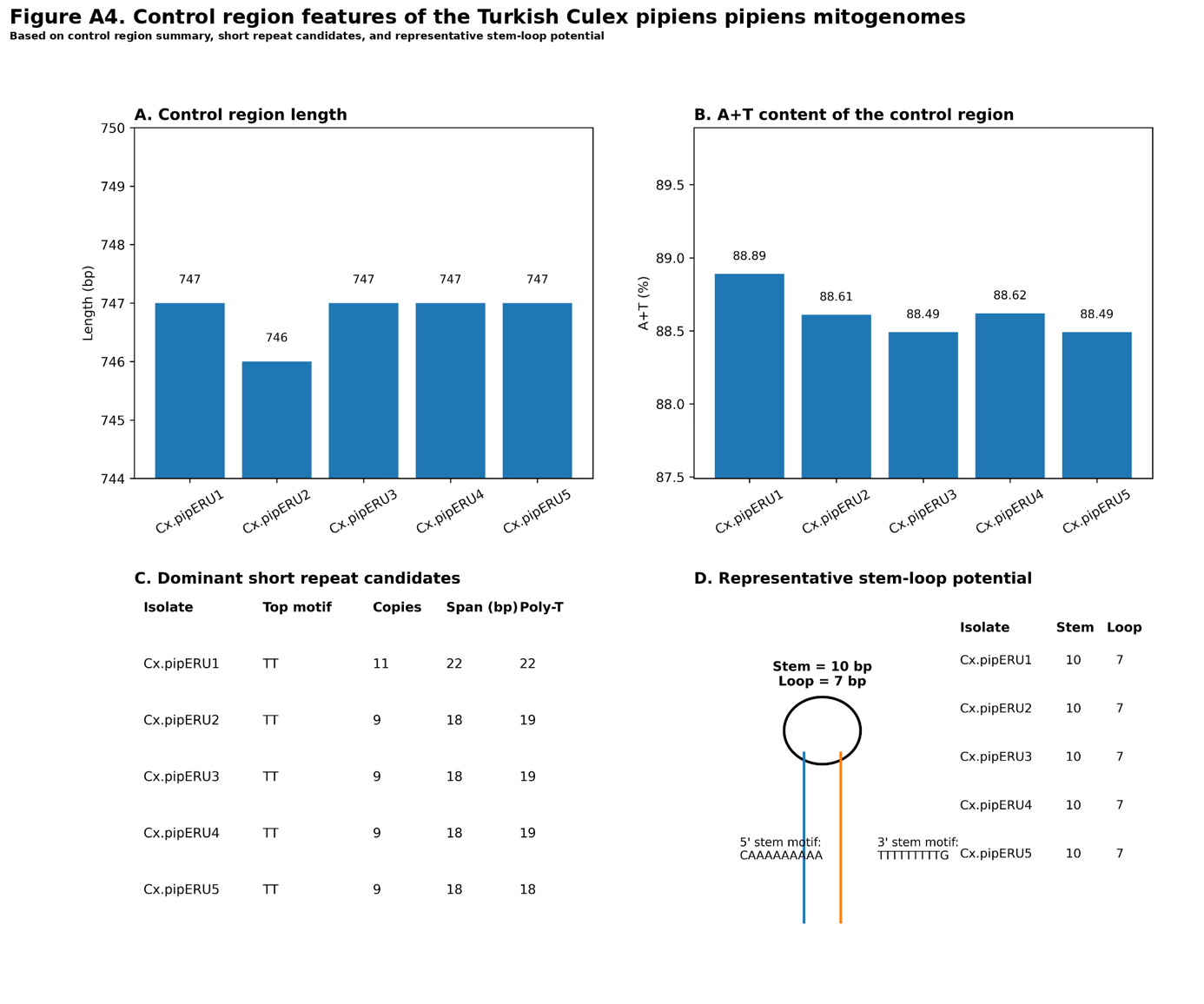
**

**Figure A4.** Control region features of the Turkish *Culex pipiens* form pipiens mitogenomes. Based on control region summary, short repeat candidates, and representative stem-loop potential.
