# Supplementary material for: Mitogenomics of Culex pipiens Form Pipiens From the Turkish Black Sea Region Reveals Structural Conservation and Phylogenetic Complexity
Source: Ecol Evol. 2026 Aug 2;16(8):e74085. doi: 10.1002/ece3.74085 (PMC13429355; doi:10.1002/ece3.74085)
Supplement: Supplementary file 2 — Table S1: Sequencing output, mitochondrial read mapping, and coverage summary statistics for the five Turkish Culex pipiens form pipiens isolates after remapping raw paired‐end reads to the common reference mitogenome NC_015079. Table S2: Feature‐based sequence variation across the broader Cx. pipiens complex complete mitogenome dataset. Table S3: Gene‐based nucleotide diversity across the broader Cx. pipiens complex dataset. [file ECE3-16-e74085-s002.docx]

**Appendix Tables**

**Table A1.** Sequencing output, mitochondrial read mapping, and coverage summary statistics for the five Turkish *Culex pipiens* form pipiens isolates after remapping raw paired-end reads to the common reference mitogenome NC_015079.

| **Short ID** | **Isolate label** | **Total reads / fragments** | **Common reference** | **Mapped reads** | **Mapped percent (%)** | **Reference length** | **Mean depth** | **Median depth** | **Min depth** | **Max depth** | **Positions covered >=1x** | **Positions covered >=10x** | **Positions covered >=100x** |
| --- | --- | --- | --- | --- | --- | --- | --- | --- | --- | --- | --- | --- | --- |
| ERU1 | Cx.pipERU1 | 28,527,453 | NC_015079 | 6,209 | 0.022 | 14,856 | 54.21 | 50.0 | 2 | 128 | 14,856 | 14,780 | 591 |
| ERU3 | Cx.pipERU2 | 58,073,455 | NC_015079 | 16,285 | 0.028 | 14,856 | 153.89 | 150.0 | 62 | 392 | 14,856 | 14,856 | 14,511 |
| ERU4 | Cx.pipERU3 | 74,206,711 | NC_015079 | 47,979 | 0.065 | 14,856 | 382.57 | 378.0 | 181 | 652 | 14,856 | 14,856 | 14,856 |
| ERU6 | Cx.pipERU4 | 51,746,081 | NC_015079 | 18,054 | 0.035 | 14,856 | 165.55 | 160.0 | 69 | 439 | 14,856 | 14,856 | 13,712 |
| ERU9 | Cx.pipERU5 | 43,379,389 | NC_015079 | 14,841 | 0.034 | 14,856 | 141.75 | 135.0 | 60 | 357 | 14,856 | 14,856 | 13,081 |

**Note:** Total reads/fragments indicate the total sequencing output summarized for each sample before mitochondrial read mapping. Mapped percent indicates the proportion of total reads/fragments that mapped to the common mitochondrial reference NC_015079.

**Table A2.** Feature-based sequence variation across the broader *Culex pipiens* complex complete mitogenome dataset.

| **Feature type** | **Gene** | **Start** | **End** | **Length (bp)** | **Variable sites** | **Constant sites** | **SNP-like columns** | **Indel-containing columns** | **Pi** |
| --- | --- | --- | --- | --- | --- | --- | --- | --- | --- |
| tRNA | tRNA-Ile | 2 | 69 | 68 | 1 | 67 | 1 | 0 | 0.001961 |
| tRNA | tRNA-Gln | 70 | 138 | 69 | 0 | 69 | 0 | 0 | 0.000000 |
| tRNA | tRNA-Met | 142 | 210 | 69 | 2 | 67 | 2 | 0 | 0.003865 |
| CDS | ND2 | 211 | 1233 | 1023 | 27 | 996 | 27 | 0 | 0.004450 |
| tRNA | tRNA-Trp | 1235 | 1303 | 69 | 0 | 69 | 0 | 0 | 0.000000 |
| tRNA | tRNA-Cys | 1304 | 1369 | 66 | 0 | 66 | 0 | 0 | 0.000000 |
| tRNA | tRNA-Tyr | 1382 | 1447 | 66 | 0 | 66 | 0 | 0 | 0.000000 |
| CDS | COX1 | 1446 | 2982 | 1537 | 64 | 1473 | 64 | 0 | 0.006296 |
| tRNA | tRNA-Leu | 2983 | 3049 | 67 | 32 | 35 | 32 | 0 | 0.068657 |
| CDS | COX2 | 3055 | 3739 | 685 | 6 | 679 | 6 | 0 | 0.001474 |
| tRNA | tRNA-Lys | 3740 | 3810 | 71 | 17 | 54 | 17 | 0 | 0.031925 |
| tRNA | tRNA-Asp | 3821 | 3888 | 68 | 0 | 68 | 0 | 0 | 0.000000 |
| CDS | ATP8 | 3898 | 4050 | 153 | 0 | 153 | 0 | 0 | 0.000000 |
| CDS | ATP6 | 4044 | 4724 | 681 | 1 | 680 | 1 | 0 | 0.000196 |
| CDS | COX3 | 4724 | 5511 | 788 | 10 | 778 | 10 | 0 | 0.002901 |
| tRNA | tRNA-Gly | 5512 | 5578 | 67 | 25 | 42 | 25 | 0 | 0.049751 |
| CDS | ND3 | 5576 | 5930 | 355 | 1 | 354 | 1 | 0 | 0.000376 |
| tRNA | tRNA-Arg | 5931 | 5994 | 64 | 0 | 64 | 0 | 0 | 0.000000 |
| tRNA | tRNA-Ala | 5995 | 6060 | 66 | 0 | 66 | 0 | 0 | 0.000000 |
| tRNA | tRNA-Asn | 6061 | 6127 | 67 | 0 | 67 | 0 | 0 | 0.000000 |
| tRNA | tRNA-Ser | 6126 | 6190 | 65 | 1 | 64 | 1 | 1 | 0.002083 |
| tRNA | tRNA-Glu | 6198 | 6263 | 66 | 1 | 65 | 1 | 0 | 0.003752 |
| tRNA | tRNA-Phe | 6262 | 6328 | 67 | 0 | 67 | 0 | 0 | 0.000000 |
| CDS | ND5 | 6329 | 8074 | 1746 | 23 | 1723 | 23 | 0 | 0.002465 |
| tRNA | tRNA-His | 8072 | 8137 | 66 | 0 | 66 | 0 | 0 | 0.000000 |
| CDS | ND4 | 8138 | 9480 | 1343 | 7 | 1336 | 7 | 0 | 0.000865 |
| CDS | ND4L | 9474 | 9770 | 297 | 0 | 297 | 0 | 0 | 0.000000 |
| tRNA | tRNA-Thr | 9776 | 9841 | 66 | 0 | 66 | 0 | 1 | 0.000000 |
| tRNA | tRNA-Pro | 9842 | 9907 | 66 | 0 | 66 | 0 | 0 | 0.000000 |
| CDS | ND6 | 9913 | 10428 | 516 | 1 | 515 | 1 | 0 | 0.000258 |
| CDS | CYTB | 10428 | 11562 | 1135 | 15 | 1120 | 15 | 0 | 0.002685 |
| tRNA | tRNA-Ser | 11563 | 11628 | 66 | 15 | 51 | 15 | 1 | 0.030336 |
| CDS | ND1 | 11647 | 12603 | 957 | 4 | 953 | 4 | 0 | 0.001174 |
| tRNA | tRNA-Leu | 12600 | 12664 | 65 | 2 | 63 | 2 | 0 | 0.004103 |
| rRNA | 16S ribosomal RNA | 12668 | 14000 | 1333 | 16 | 1317 | 16 | 3 | 0.001687 |
| tRNA | tRNA-Val | 14001 | 14072 | 72 | 2 | 70 | 2 | 0 | 0.003704 |
| rRNA | 12S ribosomal RNA | 14073 | 14857 | 785 | 8 | 777 | 8 | 0 | 0.002184 |
| Control region | Control region | 14858 | 15604 | 747 | 67 | 680 | 0 | 747 | 0.039409 |

**Note:** Gene coordinates are shown according to the representative Turkish *Culex pipiens* form pipiens mitogenome Cx.pipERU1 (PZ220808). The broader dataset included 15 mitogenomes: the five Turkish isolates and representative GenBank-derived mitogenomes of *Cx. pipiens* form pipiens, *Cx. pipiens* form molestus, *Cx. pallens*, and *Cx. quinquefasciatus*. Nucleotide diversity (Pi) values were calculated across aligned homologous regions. Columns containing gaps in at least one sequence were counted as indel-containing columns.

**Table A3.** Gene-based nucleotide diversity across the broader *Culex pipiens* complex dataset.

| **Gene** | **Start** | **End** | **Length (bp)** | **Variable sites** | **Constant sites** | **SNP-like columns** | **Indel-containing columns** | **Pi** |
| --- | --- | --- | --- | --- | --- | --- | --- | --- |
| COX1 | 1446 | 2982 | 1537 | 65 | 1477 | 63 | 41 | 0.006382 |
| ND2 | 211 | 1233 | 1023 | 26 | 997 | 26 | 0 | 0.004320 |
| COX3 | 4724 | 5511 | 788 | 10 | 779 | 10 | 1 | 0.002900 |
| CYTB | 10428 | 11562 | 1135 | 15 | 1122 | 15 | 2 | 0.002684 |
| ND5 | 6329 | 8074 | 1746 | 23 | 1732 | 23 | 12 | 0.002467 |
| 12S rRNA | 14073 | 14857 | 785 | 8 | 797 | 8 | 20 | 0.002183 |
| COX2 | 3055 | 3739 | 685 | 6 | 681 | 6 | 2 | 0.001474 |
| 16S rRNA | 12668 | 14000 | 1333 | 14 | 1359 | 9 | 64 | 0.001371 |
| ND1 | 11647 | 12603 | 957 | 4 | 953 | 4 | 15 | 0.001180 |
| ND4 | 8138 | 9480 | 1343 | 7 | 1337 | 7 | 1 | 0.000865 |
| ND3 | 5576 | 5930 | 355 | 2 | 355 | 2 | 5 | 0.000751 |
| ND6 | 9913 | 10428 | 516 | 1 | 518 | 1 | 3 | 0.000258 |
| ATP6 | 4044 | 4724 | 681 | 1 | 680 | 1 | 0 | 0.000196 |
| ATP8 | 3898 | 4050 | 153 | 0 | 162 | 0 | 9 | 0.000000 |
| ND4L | 9474 | 9770 | 297 | 0 | 300 | 0 | 3 | 0.000000 |

**Note:** Gene coordinates are shown according to the representative Turkish *Culex pipiens* form pipiens mitogenome Cx.pipERU1 (PZ220808). The broader dataset included 15 mitogenomes: the five Turkish isolates and representative GenBank-derived mitogenomes of *Cx. pipiens* form pipiens, *Cx. pipiens* form molestus, *Cx. pallens*, and *Cx. quinquefasciatus*. Nucleotide diversity (Pi) values were calculated from aligned homologous loci. Columns containing gaps in at least one sequence were counted as indel-containing columns.
